# Encapsulation of propolis extracted with methylal in the chitosan nanoparticles and its antibacterial and cell cytotoxicity studies

**DOI:** 10.1186/s12906-024-04472-8

**Published:** 2024-04-19

**Authors:** Akbar Vaseghi, Reza Ashrafi Parchin, Kosar Rezaee Chamanie, Marc Herb, Hajar Maleki, Majid Sadeghizadeh

**Affiliations:** 1 Department of Nanobiotechnology, Faculty of Biological Sciences, Tarbiat Modares University, Tehran, Iran; 2 Excir Faravaran Sabalan Company, Ardabil Science and Technology Park, Ardabil, Iran; 3 Department of Biology, Faculty of Science, University of Guilan, Rasht, Iran; 4 Institute for Medical Microbiology, Immunology and Hygiene, Faculty of Medicine, University Hospital Cologne, University of Cologne, Cologne, 50935 Germany; 5 Department of Chemistry, Institute of Inorganic Chemistry, University of Cologne, Cologne, 50939 Germany; 6 Center for Molecular Medicine Cologne, CMMC Research Center, Cologne, 50931 Germany; 7 Department of Molecular Genetics, Faculty of Biological Sciences, Tarbiat Modares University, Jalal AleAhmad St, Tehran, Iran

**Keywords:** Propolis, Methylal, Chitosan NPs, Antibiotics, Biofilm, Cell proliferation

## Abstract

In this study we develop novel type of antibacterial chitosan-propolis NPs to improve theantimicrobial activity against various pathogens. To this aim, we primarily extracted propolis with methylal and ethanol as green solvents and its encapsulation with chitosan NPs. The developed propolis loaded chitosan NPs indicated antimicrobial and anti-biofilm properties against various gram positive and negative*.* FTIR revealed the successful encapsulation of the propolis extract with Ethanol (PE) and Methylal (PM) into the chitosan nano career matrix. HPLC and GC-MASS also confirmed the presence of flavonoids and phenols compounds of propolis extracted with both solvents. In addition, we confirmed the total phenolic and flavonoid compounds in propolis by calorimetric method of Folin–Ciocalteu and aluminum trichloride complex formation assays, respectively. PE-CH and PM-CH were optimized regarding physicochemical properties such as particle size, zeta potential, and poly dispersity index (PDI) index. DLS and SEM micrographs confirmed a spherical morphology in a range of 360–420 nm with Z potential values of 30–48 mV and PDI of 0.105–0.166 for PE-CH and PM-CH, respectively. The encapsulation efficiency was evaluated using colorimetric analysis, with median values ranging from 90 to 92%. The MIC values within the range of 2 to 230 µg/ml and MBC values between 3 to 346 μg/ml against both gram-positive and negative bacteria. While both PE and PM showed a significant reduction in the number of *E. coli, S. aureus, and S. epidermidis*, the use of PE-CH and PM-CH led to a statistically significant and greater reduction in number of *E. coli, S. aureus, and S. epidermidis* strains on the biofilm, pre-formed biofilm and planktonic phases. Besides, the DPPH assay showed significant antioxidant activity for these NPs within the range of 36 to 92%. MTT assay for MHFB-1, HFF, L929, MDF, and MCF-7 cells exhibited statistically significant differences in each other that show the IC50 between 60–160 µg/ml for normal cells and 20 for cancer cells. Finally the present study indicated that both PM and PM-CH greater than PE and PE-CH in which contain high flavonoid and phenolic contents with a high antioxidation potential antioxidant properties, which could be beneficial for cell proliferation and antibiotic and anticancer applications.

**Supplementary Information:**

The online version contains supplementary material available at 10.1186/s12906-024-04472-8.

## Introduction

Drug delivery systems using nanocarriers provide an improved drug stability, effectiveness, and deep penetration [1, 2]. The choice of the nano carrier is critical as it may have an impact on the medication's or the natural active ingredient's protection, retention, and bioavailability of chosen drugs. Due to its non-toxic and non-immunogenic activities, chitosan biopolymer based nanocarrier is ideal for biomedical and pharmaceutical applications. As a nano-carrier, chitosan has the ability to encapsulate and protect drugs and other bioactive compounds, allowing for targeted delivery to specific cells or tissues. Chitosan offers the best nano carrier for natural products due to its ability for encapsulation of multi-component. Deacetylation, molecular weight, pH, ionic strength, and non-aqueous solvents all impact chitosan's antibacterial, biodegradability, and biocompatibility [3]. Microbial mortality results from interactions between the cationic chitosan and its amino group and the negatively charged cell surface of microorganisms. Therefore, chitosan combination with other antibacterial compounds, especially natural antibiotics like propolis, is an emerging concept for a successful therapy [4, 5]. The development of new antibiotics has slowed down in recent years, making it more challenging to treat infections caused by antibiotic-resistant bacteria. One interesting optionfight bacterial infections would be to use natural antibiotic solutions that have the capacity to inhibit biofilm development without bacterial resistance. Natural antibiotics have antimicrobial properties serving as potential alternatives to pharmaceuticals which prove ineffective against widespread antibiotic resistance in various industries. The resinous material such as propolis, which is gathered by honeybees, contains more than 300 distinct substances, including resins (50%), wax (30%), balms (10%), essential oils and aromatics (5%), pollen, polyphenols, flavonoids, and active ingredients (5%). Some of useful compounds in propolis are, cinamic, amino acids, benzoic acid derivatives, flavonoids (galangin, chrysin, pinocembrin), polyphenols, vitamins, carotenoids, sterols, and terpenes [6, 7]. Propolis color, smell, and chemical composition depend on the location, climate, time of collection and the type of bee species [8]. Propolis has proven with antibiotic, antioxidative, anti-inflammatory with eliminating IL-1 beta and IL-6, anti-prostatitis, anti-hepatotoxic, and anti-anemic properties. [7, 9], Besides propolis possesses antiviral activity against influenza virus type A and B, HSV-1, HSV-2, Adenovirus, HIV, infectious bursal disease virus, and avian reovirus, bovine rotavirus, bovine viral diarrhea virus, Newcastle virus, feline calicivirus, pseudorabies virus, and canine adenovirus type 2 [10], antifungal [11],as well as wound healing, and anti-cancer properties [12]. Propolis has antibacterial properties that can be used to reduce bacterial viability, interfere with bacterial growth, and prevent bacterial adhesion by inhibition of glucosyltransferase enzymes (GTFs) [9] and affect the gene expression [13]. Moreover, propolis has been used in certain instances to combat several murine and human melanoma cell lines, including B16 and A375 with reduction of NF-KB and JAK2 / STAT3, and blocking PAK1 [14]. Despite the extensive therapeutic potential of propolis, its formulation is often hindered due to its complex multi-component nature, resinous and adhesive consistency, limited solubility, and physical instability. On the other hand, due to propolis astringency and bitterness, is not as widely accepted as it could be by consumers. According to several studies, polyphenols cause those flavour characteristics and have a detrimental effect on consumer acceptability [15]. However, propolis can be encapsulated in nanoemulsions to reduce its effect on the organoleptic characteristics of the foods it is added to. Propolis's use in foods is supported by its nanoencapsulation, which improves its antimicrobial activity while lowering the impact on taste and allergy. Numerous studies have investigated the effects of different solvents on the extraction of phenolic and flavonoid compounds from propolis such as Ethyl acetate, Methanol, Dimethyl sulfoxide [16], Water [17], Chloroform, Acetone, Dichloromethane and Hexane [18]. For extracting biologically active compounds from propolis, ethanol is an appropriate solvent that is frequently utilized. [19]. Ethanol extracts of propolis have been shown to contain high level of phenolic and flavonoid compounds. Ethanol can extract these compounds effectively from propolis as it can dissolve a wide range of polar and nonpolar molecules. Phenolic compounds and their subclasses, flavonoids in propolis are known for their antioxidant properties. Chitosan-propolis nanoparticles tested against *S. epidermidis* [20]. Teik's group proposed that cationic nature in chitosan can potentially kill gram-positive bacteria biofilm with changing the zeta potential of the biofilm [20]. Dimethoxymethane, also known as Methylal, with the molecular formula C_3_H_8_O_2_, is a commonly used organic solvent that classified as an acetal. Methylal is an essential starting material and a superior solvent in the chemical industry [21]. It is a colorless liquid with a mild, ether-like odor and is soluble in a wide range of organic solvents, including alcohols, ketones, and esters. Methylal is a polar aprotic solvent, which means it can dissolve a wide range of organic and inorganic compounds that we can find in the Ps. Methylal is commonly used as a solvent in the production of resins, coatings, and adhesives, as well as in the synthesis of pharmaceuticals, agrochemicals, and other special chemical compunds. It is also used as a fuel additive and as a solvent for oils and waxes. The most important step in extracting Ps extracts is to choose an effective and economical solvent. According to the published article the phenols in Ps can be protected using Methylal [22].This protection increases the properties of phenol in the long term. High concentrations of methyl can show some exhibited symptoms of eye and respiratory tract irritation [23]. Methylal can affect breathed in and may be absorbed through the skin. But like all chemicals, most chronic (long-term) effects result from repeated exposures to a chemical. But Methylal evaporates readily during the Ps extraction procedure. Bacteria can form biofilms on the surfaces of the devices, making them difficult to eradicate with antibiotics. It does not cause harm to its human host under normal circumstances. *S. epidermidis* is a species of Gram-positive bacteria that is part of the human skin microbiota. However, *S. epidermidis* can cause opportunistic infections in individuals with weakened immune systems or in medical settings, such as hospitals. *S. epidermidis*, as normal flora and resistance to β-lactam antibiotics that used in clinical settings, can adhere to both biotic and abiotic surfaces [24]. *S. aureus* is a species of Gram-positive bacteria that can be found on the skin and in the nasal passages of healthy individuals. *S. aureus* is also a significant human pathogen that can cause a wide range of infections. *S. aureus* infections can range from minor skin infections, such as boils and impetigo, to more serious infections, such as pneumonia, endocarditis, and sepsis. The bacterium is known to produce a number of virulence factors, including toxins, enzymes, and surface proteins, that contribute to its pathogenicity. In recent years, *S. aureus* has become a significant public health concern due to the emergence of antibiotic-resistant strains, such as methicillin-resistant *S. aureus* (MRSA). Prevention of *S. aureus* infections involves good hygiene practices, such as regular hand washing, and appropriate use of antibiotics [25]. The development of new antibiotics is also being explored, in combating antibiotic-resistant *S. aureus* [26]. *E. coli* is a gram-negative, facultative anaerobe, meaning it can grow in both aerobic and anaerobic environments. However, some strains of *E. coli* have developed resistance to antibiotics, making treatment more challenging [27]. The other gram-positive, rod-shaped, non-spore-forming bacteria *L. monocytogenes* is what causes listeriosis. Food is the usual source of contamination. *P. aeruginosa*, a kind of germ-negative bacterium, can infect the blood, the lungs (pneumonia), or other areas of the body. These bacteria are always coming up with novel strategies to evade the antibiotics that are used to treat the diseases they cause. These microorganisms may become multidrug-resistant if they acquire resistance to multiple different classes of antibiotics. Primary intestinal pathogen *S. typhimurium* can infect both people and animals. *Salmonellae* enter the intestinal epithelium after being consumed in tainted food or drink, which starts the infection process. Due to the slightly polar nature of propolis components (which typically contain multiple OH groups in their molecules), HPLC is the technique of choice for propolis component identification. In this study, we are following several objectives which include 1) for the first time extracting propolis by Methylal solvent (PM), 2) investigation of various compounds from Ps extracted with Methylal using the HPLC and GC-MAS, PE and PM encapsulation with different volumes of chitosan NPs, and analyzing the in vitro propolis release profile, 3) characterizing the morphological and physicochemical properties of Ps-NPs such as particle size, polydispersity index, zeta potential, and surface morphology, 4) analyzing the antibacterial effectiveness of chitosan-propolis NPs (PM-CH) on gram-positive and negative bacteria, and 5) disruption study of PM effect on the biofilm and pre-biofilm on the (*S. aureus, S. epidermidis, L. monocytogenes*) and three gram-negative *(E. coli, P. aeruginosa, S. typhimurium*) bacteria growth. Furthermore, maybe the antibacterial qualities, toxicity, and cell growth will be different with phenol and flavonoids extracted using various solvents. Methylal and chitosan NPs can shield to phenolic compounds and exhibit a delayed release of the drug, which may offer an improved feature of Ps at a reduced concentration. Therefore, the goal of this work is to investigate the antimicrobial and antioxidant activities as well as chemical components of propolis extracted using Metylal and create a nanoformulation for use as a natural antibiotic.

## Materials and methods

### Propolis, chemicals reagents

Propolis was purchased directly from bee farms (Exir Faravaran Sabalan Company, Ardebil, Iran). Methylal (D134651), Ethyl alcohol (459,844), Medium molecular weight chitosan with deacetylation degree (75–85%: CAS: 9012–76-4), DPPH freies Radikal (300,267), and Quercetin 95% (Q4951) were purchased from Sigma-Aldrich; sodium tripolyphosphate (TPP), Folin & Ciocalteu's phenol reagent, Glacial Acetic Acid, and Tryptic Soy Broth (105,459) were purchased from Merck (Darmstadt, Germany). Aluminiumchlorid (10,165,080), DMEM (Gibco, 11,965,084), and FBS (Gibco, 16,000,044) were purchased from Thermo fisher Scientific(USA). (3-(4,5-dimethylthiazol-2-yl)-2,5-diphenyltetrazolium bromide) (MTT) purchased from Invitrogen. All cell lines acquired from National Cell Bank of Iran (NCBI).

### Bacterial strain and culture

*S. epidermidis strain (ATCC14990), S. aureus (NCTC 8325), E.coli ( ATCC:11,775), E.coli K12( NCTC 11100), L. monocytogenes (EGD-e), S. typhimurium(SL1344), and P. aeruginosa*(Boston 41,501) were used as a standard strain in this study. All bacteria were cultured at 37˚C in Tryptic Soy Broth (TSB). Bacterial suspension of 0.5 McFarland units was standardized to be used as inoculum for the experiments. All experiments were carried out in triplicates with three independent repeats.

### Preparation of methylal extract of propolis

Propolis (300g) was mixed with 99.0% Methylal (100ml). The suspensions were stored in the darkness at room temperature (25 ^∘^C). Afar 24 h, the resulting infusion was filtrated through sterile filters. The 30% ethanol extract of propolis was obtained at 37 °C for 48 h under constant agitation in a rotary shaker at 200rpm, filtered through whitman filter paper.

### Total phenolic, flavonoid, and anthocyanin's content and phytochemical analysis of methylal extracts of propolis

The Folin-Ciocalteu assay is a colorimetric method used for the quantification of total phenolic compounds in various samples. 15μl of each extract was mixed with 10ml ultrapure water, 1ml Folin–Ciocalteu reagent, and 2 ml of a 20% sodium carbonate solution (w/v). Then the ultrapure water was added to fill up the volume to 50 ml. After 1 h of reaction at room temperature, the absorbance was measured at 760 nm,as the standard for a calibration curve, and results were expressed as Gallic acid equivalents [28]. The total flavonoids were determined by mixing 1 mL of each extract with 1 mL of aluminum trichloride in ethanol 2%. The absorbance was measured after 40 min at 430 nm. To identify and quantify the flavonoid, Quercetin standard solution was used [29]. A calibration curve of Quercetin standard was plotted and used to calculate the concentration of Quercetin presenting the extracts. The total anthocyanin from propolis extracted with methylal was placed in a dark condition for 24-h. then, centrifuged at 12,000 rpm. The absorbance was read at 550 nm wavelength [30].

### HPLC and GC-MAS analysis and standardization of propolis

HPLC and GC–MS are both powerful analytical techniques used to identify and quantify chemical compounds in a sample. HPLC is the preferred technique for the analysis of propolis constituents due to their relatively polar nature (propolis constituents typically contain multiple OH groups in their molecules). Agilent 1260 HPLC system (Agilent Technologies 1260 infinity, USA) was used to characterize the Ethanol and Methyal extracts of propolis using the standard flavonoid markers Rutin, Quercetin, Luteolin, Kaempferol, Apigenin, Catechin, Vitexin, Genestin, Myricetin, Daidzein, Naringenin, Epicatechin and Pinocembrin) and phenolic standards (purity > 99.0%) Gallic acid, Cinnamic acid, Protocatechuic acid, Hydroxybenzoic acid, Vanillic acid, Caffeic acid, Syringic acid, Ferulic acid, O-coumaric acid, as internal standards (IS) were obtained from Sigma-Aldrich. Standard solution was dissolved in 10.0 ml of ethanol HPLC. Separation was carried out with a Zorbax Eclipse-AAA column (4.6 × 150 mm, 3.5-mm particle size; Agilent Technologies, USA). Mobile phase A was aqueous buffer (25 mM Na_2_HPO_4_/ NaH_2_PO, pH 7.2)/ tetrahydrofuran (95:5, v/v) and mobile phase B was aqueous buffer (25 mM Na_2_HPO_4_/ NaH_2_PO, pH 7.2)/ methanol/acetonitrile (50:35:15, v/v/v) [31]. Each standard solution was prepared at 1mg/mL concentration with methanol. Elution was done with linear gradient of 0.05% phosphoric acid in water (pH2.5) (solvent A) and methanol (solvent B) at a flow rate of 1mL/min. However, capillary GC's unparalleled resolving power, along with the useful structural data it offers, and GC–MS have recently made a noteworthy comeback for Ps compound identification. The chemical composition of ethanol, methylal, and water were determined by GC–MS using an Agilent 7890B series Gas Chromatography (GC) combined with Agilent 5977A Series Mass Spectrometer (MS) (Santa Clara, CA, USA). The MS was operated in the EI mode (electron energy = 70 eV), scan range = 10–550 amu, and scan rate = 3.99 scans/s. The GC column was an HP-5ms fused silica capillary column with the following features: 30 m length, 0.25 mm diameter, and 0.25 μm film thickness. The carrier gas was helium with a column head pressure of 53.1 kPa and a flow rate of 1.0 mL/min. Inlet temperature was 280° C, and interface temperature was 280°C. The GC oven temperature program was used as follows: 50°C initial temperature, hold for 1 min; increased at 8°C/min to 100°C; increased at 6°C/min to 110°C, hold for 1 min; then at 6°C/min to 310°C, hold for 1 min A 1% w/v solution of propolis extracted sample in methanol as solvent was prepared, and 1% μL was injected under split less mode. The propolis extracted components were tentatively identified by comparing mass spectral fragmentation patterns and retention indices (RI) based on a series of homologous C_8_–C_20_ *n*-alkanes with those reported in databases [32].

### Antioxidant activity

The antioxidant activity was evaluated based on radical scavenging properties of the propolis extract by the 2,2-diphenyl-1-picrylhydrazyl (DPPH) assay reported by Moreira et al. [33]. Briefly, the dried propolis extracts were dissolved in absolute ethanol and methylal separation. The propolis extracts were then mixed with 3.5 mL of ethanolic DPPH solution (50 mg L^−1^). The mixture was then left to stand at room temperature for 30 min in the dark. The decrease of DPPH radical in the mixture, as indicated by the reduction of its purple color, was quantified by measuring the absorbance of the mixture at 517 nm using a single beam UV–vis spectrophotometer with ethanol acting as a blank. Radical scavenging activity (RSA) of the propolis particles was determined using the following Eq. (1): where A-control and A-sample is the absorbance of mixture without and with the propolis particles, respectively.

1 $${\text{RSA}}\left(\mathrm{\%}\right)=\left[1-\frac{{{\text{A}}}_{{\text{sample}}(517\mathrm{ nm})}}{{{\text{A}}}_{{\text{control}}(517\mathrm{ nm})}}\right]\times 100$$

### Preparation of chitosan-propolis nano particles

Chitosan NPs were synthesized according to the methodology proposed by ionic gelation method [34]. Briefly, different concentrations of chitosan solutions (0.1–1%w/v) were prepared in 0.1% v/v glacial acetic acid and filtered .The specific parameters such as different concentrations of chitosan solution are evaluated to the effect of its concentration on the final size of nanoparticles. In deionized water, sodium tripolyphosphate solution (TPP) (0.2% w/v) was prepared. PE and PM extract (0.4mg/mL) were added to the to chitosan solution in a volumetric ratio of 1:1 or 1:3 (0.1–1%w/v) containing 0.4% w/v of Tween 80 under continuous stirring to produce various chitosan-propolis NPs formulations. After five minutes of sonication, the mixture was continuously stirred as TPP solution was added dropwise. Throughout the trial, the chitosan:TPP solution ratio was kept at 2:1. The chitosan-propolis conjugated nanoparticles were separated from the solution by ultra-centrifugation at 25,000 rpm for 20 min. The nanoparticles were then subjected to different characterizations.

### Characterization methods

Fourier-transform infrared spectroscopy (FTIR) analyses (Thermo Scientific Nicolet NICOLET IR100) have been performed to investigate the chemical reactions between propolis and chitosan and to provide information about the functional groups, chemical bonds, and degree of interaction between the compounds. Formulations, PE, PE-CH, PM, PM-CH, and chitosan were dried for 1h before FTIR spectrometer;. Scanning Electron Microscopy (SEM) (XL30 model, Philips, Netherlands) was conducted to confirm the particles morphology, and approximate size of nanoparticles in solid state after 24 h drying at room temperature. The nanoparticles were fixed and covered by a gold film.The nanoparticles average size (nm) was calculated using the Rulers software.The average particle size, zeta potential, PDI, and stability of the emulsion was measured by Dynamic Light Scattering (DLS) analysis by photon correlation spectroscopy (Malvern Instruments Ltd, USA).

### Encapsulation efficiency

The encapsulation efficiency (EE %) was determined with colorimetric analysis of the flavonoid and also measuring the amount of PE, PM, PE-CH, and PM-CH by Folin-Ciocalteu assay, aluminum trichloride, and HPLC reported by [35]. In order to determine the encapsulation efficiency, the PM-CH samples were firstly separated by centrifugation (20,000 rpm) for 30 min. The supernatant was removed and 1 μL of ethanol was added to the tube, vortexes well, and centrifuged for another 10 min. 100 μL of 10% aluminum chloride alcoholic solution was added to 100 μL of the supernatant, and absolute alcohol was added to obtain a final volume of 2 mL; then, The supernatant was collected and its absorption was measured by UV/Vis spectrophotometer at 410 nm. Using the calibration curve obtained in a range of 1–6 ppm of PM (absorbency = 0.2855 concentration -0.0928, R2 = 0.97), the concentrations of the PM in supernatants were estimated.

### Release of propolis from PM-CH and PE-CH

PM-CH and PE-CH was dialyzed with a 10 kDa molecular weight cut off in 200mL of phosphate buffer saline (pH7.4) at 37°C under constant stirring. The dialysis buffer was every two hours sampled periodically and analyzed propolis released. The amount of propolis released was quantified for total flavonoids and phenol contents, with UV–vis spectroscopy at 760 and 550 nm for compounds quantification, measured as Quercetin and Galic acid, as reference or representative compounds in propolis, respectively.

### Assessment of antimicrobial activity

#### Broth micro dilution assay

For CFU determination, three gram-positive (*S. aureus, S. epidermidis, L monocytogenes*) and three gram-negative (*E. coli, P. aeruginosa, S. typhimurium*) bacteria incubated in their respective media in Snap-Cap-Tubes overnight and added the same volume (so a 1:1 dilution) of PE, PE-CH,PM, and PM-CH compounds. In the untreated tubes, PBS was just added. The Minimum Inhibitory Concentration (MIC) is commonly used in microbiology and clinical settings as the lowest concentration of a drug or antibiotic agent that completely inhibits the growth of the microbe. Also, The Minimum Bactericidal Concentration (MBC) is the lowest concentration of a drug or antibiotic agent required to kills 99.9% of the bacterial population. There are a few methods for measuring the MIC and MBC such as broth dilution, agar dilution, and time-kill assay methods, which have their advantages and disadvantages, and the choice of method, will depend on the specific bacterial strain being tested and the antimicrobial agent being used. The MIC and MBC were determined using a broth microdilution method in accordance with the Clinical and Laboratory Standards Institute(CLSI) broth micro dilution method [36]. For the examination of the exponential growth phase, a suspension of each bacterium was made in Mueller–Hinton broth and incubated for 10 to 12 h at 37 °C. It was then modified using the 0.5 McFarland scale and diluted in broth to produce 5 × 10^5^CFU/mL. Depending on when each bacterium entered its exponential phase, it was incubated for 3–4 h. The MBC test is typically performed after the MIC test. Broth micro dilution of the PE, PM, PE-CH, and PM-CH nanocomposite assay were used to evaluate the MIC and MBC by using 96-plat well again *S. epidermidis*, *S. aureus*, and *E. coli* bacteria which were added directly to the liquid culture medium. Bacterial suspensions were added to each well to a final volume of 200μL and incubated at 37˚C for overnight. The antibacterial properties was calculated by measuring the absorbance (600 nm) in the micro plat reader during the exponential phase. Untreated negative controls were included. The positive controls with available antibiotics are such as Ampicillin (AMP), Penicillin (PENG), Ciprofloxacin (CIP), Clindamycin (CLI), and Vancomycin(VAN).

#### Biofilm and pre-formed biofilm assay

To determine whether and to what extent the antimicrobial activity of films coated with the examined compounds is dependent on their concentration, a range of concentrations of the extracts was investigated. The tested concentrations were established using information from articles and earlier research. Biofilm and Pre-formed biofilm assays are a method used to determine the ability of an antimicrobial agent to prevent the formation of a biofilm grown on a surface, as the surface is treated with the antimicrobial agent [37]. Crystal violet, Tetrazolium salt, live/dead staining, Confocal Laser Scanning Microscopy (CLSM), and Colony counting assays are commonly used in research and clinical settings to evaluate the effectiveness of drugs and antimicrobial agents against biofilms. The colony counting assay was used to quantify the number of viable bacteria remaining in the biofilm after exposure to three concentrations (100, 200 or 300μg/mL) of PE, PM, PE-CH, and PM-CH. In this method, bacterial suspensions of 0.5 McFarland units were prepared; 1 ml of this suspension was added into 24-well microliter plates (Eppendorf, Hamburg, Germany) to be treated with different concentrations of PE, PM, PE-CH, and PM-CH (100, 200 or 300μg/mL) incubated at 37˚C for 24 h at 150 rpm for biofilm formation. The plates, after 24 h, were washed with saline in order to remove the planktonic bacteria. The supernatant planktonic bacteria and biofilm bacteria were serially diluted and plated on Tryptic Soy Agar. Untreated bacteria were used as negative control. [20]. For Pre-formed biofilm assay prepared as mentioned above; 1 ml of bacteria suspension was added to treat in 24-well microliter plates and incubated at 37˚C for 16 h at 150 rpm to facilitate the formation of biofilm. After 16 h, the plate was washed with saline containing PE, PM, PE-CH, and PM-CH (100, 200 or 300μg/mL) was added to the wells and treated for 8 h. After incubation, the planktonic bacteria were removed and the plate was washed with saline. The number of bacteria present in planktonic was enumerated as described above. All data are represented in both Biofilm and pre-formed biofilm assay of three independent experiments.

#### In vitro* cytotoxicity assay*

In this study in vitro cytotoxicity was determined using MTT assay as a quantitative, sensitive, and trustworthy colorimetric approach which determine the cell viability. The cytotoxicity was investigated using on the normal mouse fibroblast skin cells (MHFB-1), human foreskin fibroblasts ( HFF-1), mouse fibroblast cell line(L929), and mouse embryo fibroblast (NIH 3T3). Briefly, cells were seeded in 96-well plates at an initial density of 5 × 10 ^3^ cells/well. Cells were cultured in DMEM (Dulbecco's Modified Eagle's Medium) medium containing 10% fetal bovine serum (FBS), and penicillin (100 U/mL) at 37 C in a humidified atmosphere of 5% CO_2_. The samples PE, PM, PE-CH, and PM-CH were diluted with medium to prepare five concentrations of each sample (200,150,100, 25, 6.25, 1.56 and 0.39 g /mL) and were added to the cells mentioned above. Drug-free (blank) was used as controls to eliminate the cytotoxic effect of the PM-CH. After incubation at 37C for 24, 48, and 72 h, MTT reagent was added (50 μL of 5 mg/mL MTT solution in DMSO), and cells were further incubated at 37 C for 4 h. The absorbance of each well was read at 570 nm using a micro-plate reader (Bioline Elisa plate reader, Maharashtra, India) according to the manufacturer’s instructions. All tests were performed with three independent experiments.

#### Statistical analysis

Statistical comparisons of groups were analyzed by Graph Pad Prism 8 (Graph Pad Software, San Diego, CA, USA) one-way ANOVA followed by Tukey’s post-hoc test. Comparisons between treatments were considered significant when *P* value was>0. 05. All experiments were carried out in triplicate.

## Results

### Composition of propolis

The composition of propolis extracted using different solvents can vary in terms of the types and concentrations of bioactive compounds present. Propolss is mainly extracted using alcohol, water, dimethyl sulfoxide (DMSO), dichloromethane (DCM), Hexane, and Supercritical fluid. Hydroalcoholic extraction, such as ethanol (PE) and Methanol (MeEP), is commonly used to extract and analyze Ps [12]. Propols extracts (30% w/v) were prepared using 70% PE and PM, respectively. The greatest yield percentage (30–40%) was linked to ethanol and metylal, according to the extraction yield (mass of extract/mass of dried material) percentage. The PE and PM extraction tends to yield a propolis extract with high concentrations of flavonoids, phenolic acids, and terpenes, while water extraction tends to yield a propolis extract with high concentrations of water-soluble compounds, such as polysaccharides and amino acids. Because the main components of propolis are more soluble in polar solvents like metylal than in ethanol, this result is consistent with PM's idea concerning the polarity difference between the solvents used [38].

### Total phenolic, favonoids of methylal extracts of propolis

The amount of biologically active values for propolis was determined by quantifying total phenolics and flavonoids. However, tests for the content of flavonoids and phenols may not always accurately reflect antimicrobial activity in vitro. The determined equation of the calibration curve from Gallic acid was y = 0.0029x + 0.0184 with correlation coefficient of 0.99. The total phenolic content in the PM, PM-CH, PE and, PE-CH sample were 1361, 152, 1180, and 142.8 μg/ml respectively. Also, the equation of the calibration curve from Quercetin was y = 0.0007x + 0.0112 with a correlation coefficient equal to 0.90. The total flavonoid content in PM, PM-CH, PE, and PE-CH samples were 5628, 620, 4103, and 581 μg/ml, respectively. Since Methylal is a polar solvent that can dissolve a variety of phenolic and flavonoids compounds from Ps. Our results confirm that the Metylal is effective for extracting a total phenol and flavonoids from propolis.

### HPLC analysis of propolis extracts

The Folin-Ciocalteu assay is an effective method for estimating total phenolic content in propolis, but it does not reveal the precise phenolic chemicals present in the sample. Therfore, we employed HPLC in conjunction with the Folin-Ciocalteu assay to identify and quantify certain phenolic compounds in order to further examine all the relevant substances. The extracts were analyzed by HPLC using eleven flavonoids (Vetexin, Epicatechin, Rutin, Ginestine, Diosmin, Apigenin, Catechin, Myricetin, Luteolin, Kaempferol, Quercetin, Diadzein, and Narengenin) and ten phenols (Gallic acid, Caffeic, Chlorogenic, Hydroxybenzoic, Coumaric, Ferulic, Rosmari, Sinapic, Cinnamic, Resveratrol, and Salicylic acid) as standard markers. For these standard markers, linearity of each standard line from 0.1 μg/mL to 10 μg/mL was evaluated. The correlation coefficient to observe ranged from 0.9954 to 0.9989. The representative HPLC profile of PM and PE extracts of propolis and also the standard flavonoid and phenol markers are shown in Figs. S1 and 2 respectively. Since PM is more polar than PE, the total flavonoid concentration was greater in PM extract as compared to PE extract. The flavonoids include catechin (8.56 min), epicatechin(10.12 min), Quercetin (12.22 min), vitexin(18.58 min), rutin(21.63 min), genestin(27.35 min), myricetin(41.31 min), luteolin (44.77 min), diosmin(43.61 min), narengenin(47.00 min), apigenin(49.30 min), kaempferol (50.75 min), and pinocembrin (55.77 min) were identified to be present in PE, PM, PE-CH, and PM-CH extracted. As a flavonoid, galangin has the ability to inhibit the activity of lipo- and cyclooxygenase (COX), limit the activity of polygalacturonase, and decrease the expression of the COX-2 inducible isoform. The largest contributor to PE's total flavonoid content is pinocembrin (5,7-dihydroxyflavanone). Some phenols included gallic acid (10.38 min), caffeic acid (14.80 min), proto-catechuic, p-hydroxybenzoic (19.66 min), chlorogenic (18.90), p-coumaric (22.87 min), ferulic(28.26 min), rosmari (29.00 min), sinalic (32 min), salicylic acid (34.00 min), trans-cinnamic (37.01 min), and resveratrol (44 min) acid have been analyzed (Figs. S1 and 2, Table 1). Propolis also contains caffeic acid phenethyl ester (CAPE), which has anti-inflammatory properties by preventing the release of arachidonic acid from the cell membrane. This suppresses the activity of COX-1 and COX-2 and prevents the activation of COX-2's genic expression. The HPLC result shows a higher concentration of coumaric acid in PE and PM extracts of Ps compared with water extracts. Flavonoids among the detected flavonoids, quercetin and apigenin were present in PM and PE in substantial concentrations (11.8 and 13.3 μg/mL). As compared to PE extract, PM showed a greater quantity of Quercetin. Other flavonoids, such as Rutin, Genestin, and Pinocombrin, had modest (5.5–7.8 μg/mL) and comparable amounts in the two extracts. Chrysin, kaempferol, and quercetin have been shown in some studies to have antibacterial and anti-inflammatory properties. Resveratrol, Hydroxybenzoic, and Salicylic acid were the most prevalent phenolic acid in PM compounds. Resveratrol, a stilbene derivative, has been identified by HPLC in PE and PM. Fibronectin degradation was inhibited by the active ingredients in propolis, such as Quercetin and Resveratrol. Research has also demonstrated that a decrease in the amount of fibronectin in the extracellular matrix is necessary for the migration and mobility of epithelial cells. Propolis contains reduced levels of fibronectin, a glycoprotein that effectively heals wounds and creates granulation tissues [39]. Salicylic acid and Cinnamic displayed a range of compounds, respectively. Cinnamic, Sinalic, and Hydroxybenzoic were the most prevalent phenolic acids in PM compounds. According to HPLC records, a class of aromatic, carboxylic acids present in PM and PE are Cinnamic acid and its derivatives. By rupturing the cell membrane, Cinnamic acid and its derivatives prevent bacteria from proliferating, dividing, and forming biofilms. They also exhibited anti-quorum sensing behaviour. The retention time, regression equation, correlation coefficient of each standard, and the concentrations of the identified flavonoids in PE, PM, PE-CH, and PM-CH extracts are presented in Table 1.

**Table 1:** Calibration curve and correlation coefficients of standard flavonoids detected by HPLC

Compounds		Flavonoids						
	Retentiontime(Min)		Regressionequation	Correlation coeffcient(r^2^)	PE μg/mL	PM μg/mL	PE-CH μg/mL	PM-CH μg/mL
**Catechin**	8.2		y = 11.231x + 0.001	0.999	0.5	0.9	0.31	0.13
**Epicatechin**	10.35		y = 30.28x + 0.01	0.997	3.5	0.5	0.6	0.05
**Quercetin**	14.25		y = 64. 12x + 5	0.993	7.8	11.32	1.9	0.02
**Vitexin**	19.9		y = 39.88x + 3.1	0.9676	0.35	0.7	O.29	0.03
**Diosmin**	20.85		y = 36.493x + 5.1	0.9919	0.4	0.37	0.04	0.03
**Rutin**	21.58		y = 24.086x + 4.50	0.9954	7.8	1.2	2	0.09
**Genestin**	27.35		y = 54.06x + 0.02	0.989	5.6	0.2	0.44	0.1
**Myricetin**	41.30		y = 79.918x + 0.002	0.9983	5	1.3	0.31	0.22
**Daidzein**	44.73		y = 85.73x + 1.2	0.9858	1.2	0.11	0.27	0.06
**Luteolin**	47.04		y = 46.21x + 2.3	0.9989	3.4	0.28	0.43	0.04
**Naringenin**	49.31		y = 94.712x + 1.4	0.9817	1.7	0.15	0.19	0.024
**Apigenin**	50.33		y = 127.87x + 1.6	0.9918	13.3	0.65	0.7	0.29
**Kaempferol**	50.75		y = 111.77x + 2.9	0.9982	1.0	0.15	0.1	0.027
**Pinocombrin**	55.85		y = 111.552x + 3.3	0.9954	5.55	0.78	0.8	0.198
**Phenol**								
** Gallic acid**	10.38		y = 11.96x + 0.12	0.999	1.6	1.9	0.1	0.2
** Caffeic acid**	14.8		y = 91.557x + 5	0.9978	3.1	2	0.5	0.16
** Chlorogenic**	19.6		y = 112.90x + 2.3	0.972	1.8	1.9	0.6	0.35
** Hydroxybenzoic**	22		y = 48.86x + 5.3	0.99	5.6	9.9	0.89	0.89
** Coumaric**	22.87		y = 248.72x + 0.01	0.995	5.4	2	1.3	0.11
** Ferulic**	28.26		y = 102.42x + 2	0.9976	6.5	3.8	1.9	0.22
** Rosmari**	29.02		y = 67.16x + 0.001	0.98	2.0	4.1	0.7	0.34
** Sinalic**	34.26		y = 120.9x + 0.45	0.999	9.6	1	1.7	0.08
** Salicylic acid**	34.04		y = 27.482x + 0.2	0.9972	3.4	9.8	0.7	1.1
** Cinnamic**	37.96		y = 168.96x + 3.2	0.9977	9.1	3.1	1.9	0.22
** Resveratrol**	43.3		y = 144.02x + 4.1	0.9659	5.4	9.9	0.5	0.99

### GC–MS analysis

In our study, propolis was collected from Ardabil province, located in the north-west of Iran. GC–MS analysis of propolis extracts was carried out. About 49 and 42 unique compounds from Ethanol and Methylal solvents were identified, including aromatic acid and their related esters, carbohydrates, polymers, hydrocarbons, flavonoid and flavonoid derivatives (Flavones, Flavonols, Flavanones, Flavanonols, Chalcones, Dihydrochalcones, Isoflavones, Isodihydroflavones, Isoflavones, Neoflavonoids, and Flavonoid Glycosides), phenol, compounds such as ketone, Alkaloids, Aromatic acid, Fatty acids. Also, results showed the terpene derivatives include Dihydro, Alpha, Terpineol Acyclic, monocyclic, and dicyclic monoterpenes. The antioxidant activity of the extract may be attributed to the phenolic compounds, which are mainly recognized for their ability to scavenge free radicals. Various terpenoid compounds such as Nerolido, Nerolidol, and Nerolidol that were similarly identified with PE and PM GC-MAS results, contribute to the functional properties of propolis, including their antioxidant, antimicrobial, antitumor, and antifungal [40]. Other compounds were hydrocarbons, including alkanes, alkenes, alkadienes, monoesters, diesters, aromatic esters, fatty acids and steroids. Ps consists mainly of resins and beeswax, both of which are hydrophobic. The results obtained are summarized in Tables 2 and 3 and GC/MS profile of Fig. S3. On the other, Because propolis has a complex composition that makes simple material division challenging, just a tiny percentage of propolis compound groups could be analysed using the GC–MS technique.

**Table 2:** Chemical composition of Propolis extracted with Ethanol (PE)

COMPOUND SUMMARY	CLASSIFICATION	MOLECULAR FORMULA	SYNONYMS	PUBCHEM CID	CAS
1-Aziridineethanamine		C_4_H_10_N_2_	1-Aziridineethanamine	97,697	4025–37-0
Methylhydrazine	Methylhydrazines	CH6N2	Methylhydrazine	6061	60–34-4
Dimethyl ether	Methyl Ethers	C2H6O	Dimethyl Ether	8254	115–10-6
2-Ethyloxetane		C5H10O	2-ethyloxetane	521,218	
Ethyl acetate	Acetates	C4H8O2	Ethyl Acetate	8857	141–78-6
1,1-Diethoxyethane	Ethers	C6H14O2	Acetal	7765	105–57-7
4-Pentenyl acetate	carboxylic ester	C7H12O2	4-Pentenyl acetate	74,096	1576–85-8
D-Glucose, diethyl mercaptal		C10H22O5S2	D-Glucose, diethyl mercaptal	95,420	6748–69-2
chloromethyl-isoxazolidin-3-one		C6H8ClNO3	2-Acetyl-5-chloromethyl-isoxazolidin-3–1	536,688	
Nerolidyl acetate		C17H28O2	Nerolidyl acetate	5,363,426	2306–78-7
Nerolidol	Terpenes	C15H26O	Nerolidol	5,284,507	7212–44-4
Cedrelanol	Terpenes	C15H26O	Cedrelanol	160,799	5937–11-1
beta-Eudesmol	Terpenes	C15H26O	beta-Eudesmol	91,457	473–15-4
12,15-Octadecadiynoic acid,		C19H30O2	12,15-Octadecadiynoic acid, methyl ester	538,453	57,156–95-3
Ethyl palmitate	Palmitic Acids	C18H36O2	Ethyl Palmitate	12,366	628–97-7
2-Heptadecanone		C17H34O	2-HEPTADECANONE	18,027	2922–51-2
10-Octadecenal		C18H34O	10-Octadecenal	5,365,012	56,554–92-8
17-Octadecenal		C18H34O	17-Octadecenal	41,922	56,554–86-0
14-Octadecenal		C18H34O	14-Octadecenal	5,367,669	56,554–89-3
Z-(13,14-Epoxy)tetradec-acetate		C16H28O3	Z-(13,14-Epoxy)tetradec-11-en-1- acetat	5,363,633	
12-Methyl-E,E-octadecadien-1-ol		C19H36O	12-Methyl-E,E-2,13-octadecadien-1-ol	90,107,969	
2-Heptadecanone		C17H34O	2-Heptadecanone	18,027	2922–51-2
2-Nonadecanone	ketone	C19H38O	2-Nonadecanone	69,423	629–66-3
5-Hydroxy-7-methoxyflavanone	Flavanones	C16H14O4	Pinostrobin	4,101,463	75,291–74-6
3',8,8'-Trimethoxy-3-piperidyl-2,2'-binaphthalene-1,1',4,4'-tetrone		C28H25NO7	SCHEMBL17650609	590,815	12,761,184–1
Ethyl acetate	Carboxylic Acids	C4H8O2	Ethyl Acetate	8857	141–78-6
1,1-Diethoxyethane	Ethers	C6H14O2	Acetal	7765	105–57-7
3-Methyl-3-buten-1-OL	Butanols	C5H10O	3-Methyl-3-Buten-1-Ol	12,988	763–32-6
3-Methyl-2-buten-1-OL	Pentanols	C5H10O	3-Methyl-2-Buten-1-Ol	11,173	556–82-1
4-Pentenyl acetate	acetate ester	C7H12O2	4-Pentenyl acetate	74,096	1576–85-8
D-Glucose, diethyl mercaptal		C10H22O5S2	D-Glucose, diethyl mercaptal	95,420	6748–69-2
Nerolidyl acetate		C17H28O2	Nerolidyl acetate	5,363,426	2306–78-7
Elaidic acid	Oleic Acids	C18H34O2	Elaidic acid	637,517	56,599–46-3
12,15-Octadecadiynoic acid		C19H30O2	12,15-Octadecadiynoic acid, methyl ester	538,453	
Ethyl palmitate	Palmitic Acids	C18H36O2	Ethyl Palmitate	12,366	628–97-7
2-Heptadecanone	Fatty Acyls	C17H34O	2-Heptadecanone	18,027	2922–51-2
10-Octadecenal		C18H34O	10-Octadecenal	5,365,012	56,554–92-8
17-Octadecenal		C18H34O	17-Octadecenal	41,922	56,554–86-0
14-Octadecenal	Fatty Acyls	C18H34O	14-Octadecenal	5,367,669	56,554–89-3
Z-(13,14-Epoxy)tetradec-ol acetate		C16H28O3	Z-(13,14-Epoxy)tetradec-11-en-1-ol acetate	5,363,633	
12-Methyl-E,E-2,13-octadecadien- C19H36O		C19H36O	12-Methyl-E,E-2,13-octadecadien-1-ol	90,107,969	
2-Heptadecanone	Fatty Acyls	C17H34O	2-Heptadecanone	18,027	2922–51-2
2-Nonadecanone	ketone	C19H38O	2-Nonadecanone	69,423	629–66-3
5-Hydroxy-7-methoxyflavanone	Flavonoids	C16H14O4	Pinostrobin	4,101,463	75,291–74-6
5-Hydroxy-7-methoxyflavanone	Flavonoids	C16H14O4	Pinostrobin	4,101,463	480–37-5
3',8,8'-Trimethoxy-3-piperidyl-2,2'-binaphthalene-1,1',4,4'-tetrone		C28H25NO7	Schembl17650609	590,815	
Phen-1,4-diol, 2,3-dimethyl-5-trifluoromethyl- C9H9F3O2		C9H9F3O2	2,3-Dimethyl-5-(trifluoromethyl)-1,4-benzenediol	590,850

**Table 3:** Chemical composition of Propolis extracted with Metylal (PM)

COMPOUND SUMMARY	CLASSIFICATION	MOLECULAR FORMULA	SYNONYMS	PUBCHEM CID	CAS
1-Aziridineethanamine		C_4_H_10_N_2_	1-Aziridineethanamine	97,697	4025–37-0
DL-Alanine	Amino Acids	C_3_H_7_NO2	Dl-Alanine	602	302–72-7
Nerolidol	Terpenes	C15H26O	Nerolidol	5,284,507	40,716–66-3
Cedrelanol	Terpenes	C15H26O	Cedrelanol	160,799	5937–11-1
beta-Eudesmol	Terpenes	C15H26O	beta-Eudesmol	91,457	473–15-4
Dihydro-beta-ionone	Terpenes	C13H22O	Dihydro-beta-ionone	519,382	17,283–81-7
Emulphor	Polyethylene Glycols	C20H40O2	Emulphor	5,364,713	5353–25-3
Octadecanal	aldehyde	C18H36O	Octadecanal	12,533	638–66-4
Nonadecatriene		C19H34O2	E,E,Z-1,3,12-Nonadecatriene-5,14-diol	5,364,768	
1-Heptatriacontanol		C37H76O	1-Heptatriacontanol	537,071	105,794–58-9
1-Chlorooctadecane	Hydrocarbons	C18H37Cl	1-Chlorooctadecane	18,815	3386–33-2
Geranyl isovalerate	Fatty esters	C15H26O2	Geranyl isovalerate	5,362,830	109–20-6
2-Pentadecanone	Ketones	C15H30O	Pentadecan-2-one	61,303	2345–28-0
2-Nonadecanone	ketone	C19H38O	2-Nonadecanone	69,423	629–66-3
Emulphor	Polyethylene Glycols	C20H40O2	Emulphor	5,364,713	5353–25-3
2-Bromooctadecanal		C18H35BrO	2-Bromooctadecanal	537,255	56,599–95-2
Ferulic acid	phenolic	C10H10O4	ferulic acid	445,858	1135–24-6
Oleic acid	Oleic Acids	C18H34O2	oleic acid	445,639	112–80-1
Erucic acid	Erucic Acids	C22H42O2	Erucic Acid	5,281,116	112–86-7
Tetracosane	Hydrocarbons	C24H50	Tetracosane	12,592	646–31-1
Tetratetracontane	solid wax	C44H90	Tetratetracontane	23,494	7098–22-8
3-Deoxyestradiol	steroid	C18H24O	3-Deoxyestradiol	228,944	2529–64-8
5-Hydroxy-7-methoxyflavanone	Flavonoids	C16H14O4	Pinostrobin	4,101,463	480–37-5
Pentacosane	Hydrocarbonsn wax	C25H52	Pentacosane	12,406	629–99-2
Androst-5,7-dien-3-ol-17-one		C19H26O2	Nsc124732	276,591	
Nalpha		C12H16N6O6	Nalpha-(2,4-Dinitrophenyl)-L-arginine	7,083,742	1602–42-2
binaphthalene-1,1',4,4'-tetrone		C28H25NO7	3',8,8'-Trimethoxy-3-piperidyl-2,2'-binaphthalene-1,1',4,4'-tetrone	590,815	
Benzyl ferulate		C17H16O4	benzyl ferulate	7,766,335	132,335–97-8
9-cis-Retinal	Carotenoids	C20H28O	9-cis-Retinal	6,436,082	514–85-2
Tectochrysin	Flavonoids	C16H12O4	Tectochrysin	5,281,954	520–28-5
Dotriacontane	Alkanes	C32H66	Dotriacontane	11,008	544–85-4
Oleic acid	Oleic Acids	C18H34O2	oleic acid	445,639	112–80-1
Arachidyl palmitoleate	Fatty esters	C36H70O2	Arachidyl palmitoleate	5,365,040	22,522–34-5
Octadecoxypropoxy)octadecane	wax	C39H80O2	1-(2-octadecoxypropoxy)octadecane	545,620	35,545–51-8
Octadecane		C26H54	3-Ethyl-5-(2-ethylbutyl)octadecane	292,285	55,282–12-7
17-Pentatriacontene	wax	C35H70	17-Pentatriacontene	5,365,022	6971–40-0
Bacteriochlorophyll-c-stearyl		C52H72MgN4O4-2	Bacteriochlorophyll-c-stearyl	5,367,801	

### Antioxidant activity using DPPH method

Antioxidant activity (AA %) was 89.57 ± 0.093 b in the PM and 92.52 ± 0.13a in the PE, respectively (*F* = 6526.43; DF = 2, 12; *P* < 0.0001). Also, our assay sows that 36.50 ± 1.12 b and 43.90 ± 0.19 a in the PE-CH and PM-CH irrespectively (*F* = 33.88; DF = 2, 6; *P* < 0.0005).In the PM, anthocyanin content in 550 nm wavelengths was 0.034 µg/ml. Our result shows PE has a higher antioxidant capacity (Table 4). These findings have previously been shown by several writers to show that propolis is an effective free radical scavenger [41, 42]. Overall, the reduction in antioxidant activity after nano encapsulation can be a complex phenomenon that depends on many factors, including the specific antioxidant compound, the encapsulating material, and the encapsulation method. Chitosan can alter the solubility of the antioxidant compound, making it less effective at scavenging free radicals. Also, chitosan may interact with the antioxidant compound, leading to changes in its activity. Chitosan with a hydrogen bond reacts with the flavonoids, reducing its availability or reactivity. (Table 4). The low antioxidant content of PE-CH and PM-CH in contrast to PE and PM may causethe sluggish release of flavonoids from the nano-carrier.

**Table 4:** Total flavonoids, phenolic, and anthocyanin content of propolis extracted with ethanol and methylal. Also, particle size, polydispersion index, and zeta potential of propolis extract-loaded nanoparticles in suspension

Compounds	Phenolic μg/ml	Flavonoids μg/ml	DPPH	Average particle size(nm)	Polydispersity index (PDI)	Zeta potential(mV)
Chitosan-TPP				**130**	**0.44**	**35.5**
PE	**1180**	**4103**	**92.52 ± 0.13 a**	**-**	**-**	**-**
PM	**1361**	**5628**	**89.57 ± 0.093 b**	**-**	**-**	**-**
PE-CH	**152**	**620**	**36.50 ± 1.12 b**	**361**	**0.166**	**30.3**
PM-CH	**142.8**	**581**	**43.90 ± 0.19**	**420**	**0.105**	**48.6**

### Characterization of chitosan-propolis nanoparticles

**Fig. 1:**
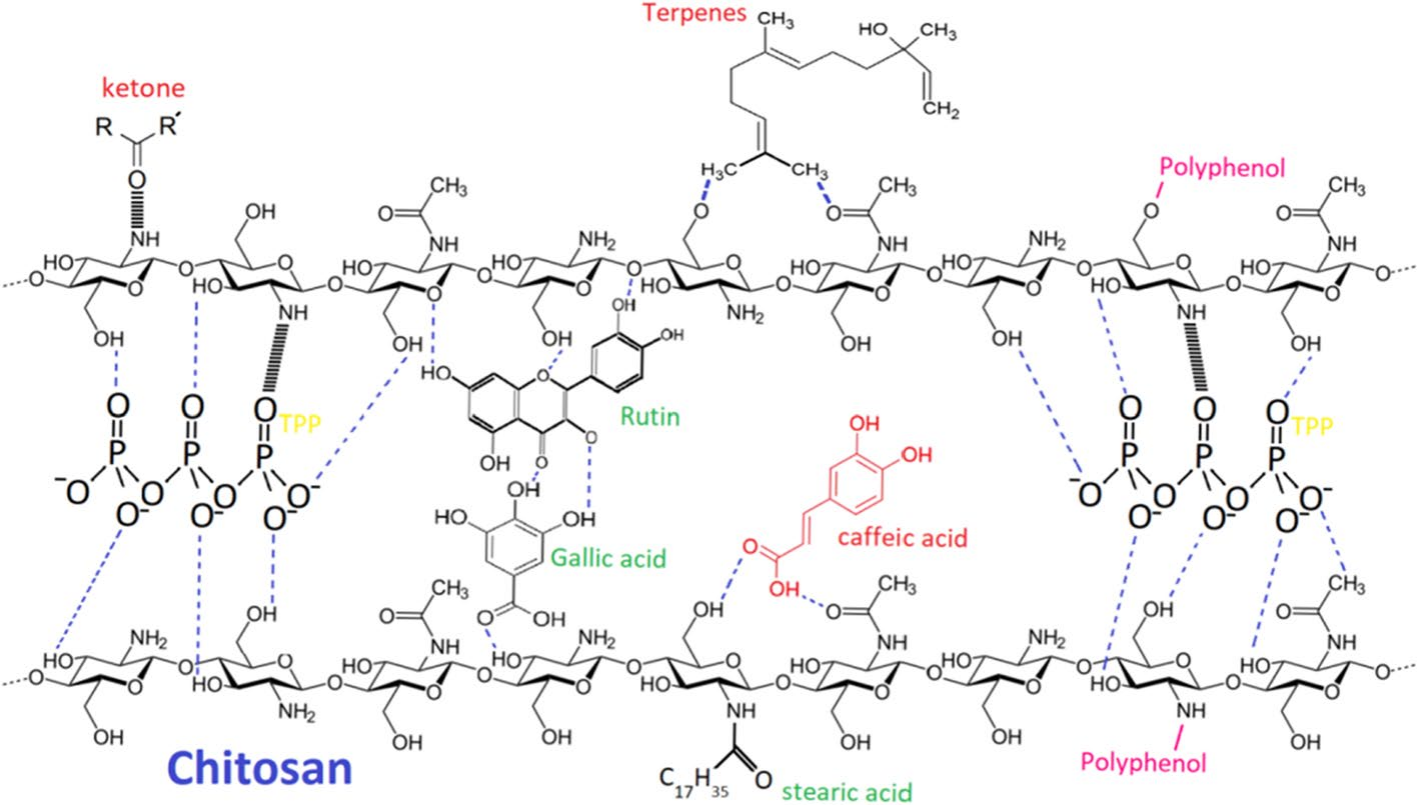
Proposed mechanism of propolis attachment with chitosan nanoparticles

The encapsulation of propolis in the chitosan nanoparticle matrix (Fig. 4) demonstrates only physical interactions between both components without involving the functional groups of PE-CH and PM-CH for covalent interactions. This is also consistent with the previously reported works [20], highlighting the propolis and chitosan interactions through only hydrogen bonds (Fig. 1). In addition to being a useful metric for assessing the stability of the nanoparticles in suspension, pH can also be used to detect drug diffusion into an aqueous medium or polymer degradation. The pH ranged from 4.6 to 6.0. observed changes in particle size, including significant rise in size, following the addition of chitosan concentration.

### FTIR analysis

The FTIR spectra of PE (A), PE-CH (A), PM (B), PM-CH (B) in Fig. S4 indicate characteristic peaks corresponding to the functional groups in the compounds, such as the amino and hydroxyl groups in chitosan and the phenolic groups in propolis. In chitosan spectrum: a characteristic band at 3347cm^−1^ due to the stretching vibration of the O–H and N–H bonds; two absorption band at 1653 and 1591 cm ^−1^, which can be attributed to amide I (C = O stretching) and N–H (amine) vibration overlapped to amide II (N–H vibration), respectively and, A band at 1380cm^−1^ can be attributed to the -CH_3_ symmetrical deformation, at 1154 cm ^−1^, two bands that can be attributed to C–O–C and C-O vibration. From PM, typical bands at 1032–1451 cm^−1^ symmetrical and asymmetric bending of the C-O and C–OH group were considered indicatives of the presence of lipids and flavonoids and flavone, phenol, attributed to aromatic ring deformations. At 1604–1636 cm^−1^, attributed to C = O stretching of flavonoids and lipids, found in PM, 3025 cm^−1^ vibrational stretch of the O–H group and N–H bonds, 2848–2910 cm^−1^ symmetrical and asymmetrical vibrations of C-H groups. The band at 2920 cm^−1^ is attributed to C–H vibration, and the presence of flavonoids was further suggested by the absorbance band at 694–754 cm^−1^ attributed to the asymmetric vibrational stretching of C–C groups of Methylal. From another solvent, PE, typical bands at 1159–1269 cm^−1^ symmetrical and asymmetric bending of the C-O and C–OH group were considered indicatives of the presence of lipids and flavonoids and flavone, phenol, attributed to aromatic ring deformations. At 1598–1674 cm^−1^, attributed to C = O stretching of flavonoids and lipids, found in PE, 3300 cm^−1^ vibrational stretch of the O–H group and N–H bonds, 2924 cm^−1^ symmetrical and asymmetrical vibrations of C-H groups. Also, The bond at 2920 cm^−1^ attributed to C–H vibration. The presence of flavonoids was further suggested by the absorbance band at 813–1081 cm^−1^ attributed to the asymmetric vibrational stretching of C–C groups of Ethanol. The spectrum of PM-CH (1028, 1264, and 1604) showed characteristic bands of both chitosan (e.g., 1034–1067 cm ^−1^) and PM (e.g., 1032, 1157, 1270, 1370, 1451, and 1604 cm^−1^), with significant shifts. Moreover, the first band of FTIR spectra was widened and shifted to a higher frequency (3347 to 3285 cm^−1^ and 2921 cm ^−1^) compared to PM-CH spectrum, suggesting the hydrogen bonding between chitosan and propolis. For the other components, PE-CH, the spectrum of PE-CH (1029, 1155, 1259, 1446, 1600, and 1638 cm^−1^) showed characteristic bands of chitosan (e.g., 1034–1067 cm ^−1^) and PE (e.g., 1027, 1159, 1269, 1598, and 1638 cm^−1^), with significant shifts. Moreover, the first band of FTIR spectra was widened and shaifted to a higher frequency compared to PM-CH spectrum from 3347 to 3300 cm ^−1^, suggesting the hydrogen bonding between chitosan and propolis. No new bands were observed in any of the PE-CH and PM-CH spectra, which demonstrated that propolis compounds interact via hydrogen bondings. Also, Because of the potential creation of hydrogen bonds between the hydroxyl groups in chitosan and the phenolic OH groups in propolis, the intensity of the absorption peaks was reduced [43] which could be ascribed to the combination of the propolis and chitosan products.

### Nanoparticle's size, zeta potential, and PDI of PM-CH

Table 4 shows particle size, polydispersity index, and zeta potential data of PE-CH and PM-CH nanoparticulate systems. Previous works reported that the size of chitosan NPs loaded with propolis ranges from 200 to 500 nm [20]. In these reported works, an increase or reduction in chitosan concentration was typically accompanied by an increase and decrase in particle size [13]. The average particle size of PE-CH and PM-CH were 420 and 361 nm with respectively. According to this point, the attainment of a stable colloidal dispersion by the repulsion between particles, which inhibits the occurrence of the nanoparticles aggregating, is represented by the high values of the zeta potential, either negative or positive (± 30 mV). The zeta potential of PM-CH, and PE-CH, due to the cationic property of the chitosan NPs, has positive charge of 48.6 and 30.3 mV respectively. Our result showed that PDI of PE-CH and PM-CH is 0.166 and 0.105, respectively, suggesting that they were homogeneous or mono dispersed particles. (Table 4 Fig. S5). Also, we evaluated the NPs the stability of during 6 months and no aggregations or precipitation was observed. The positive zeta potential values were sufficient to stabilize the chitosan NPs of PM-CH. For above mentioned results, 0.2% w/v as an ideal concentration was chosen for further evaluation of its anti-bacterial and anti-biofilm activity.

### PE-CH, and PM-CH NPs morphology by SEM

**Fig. 2:**
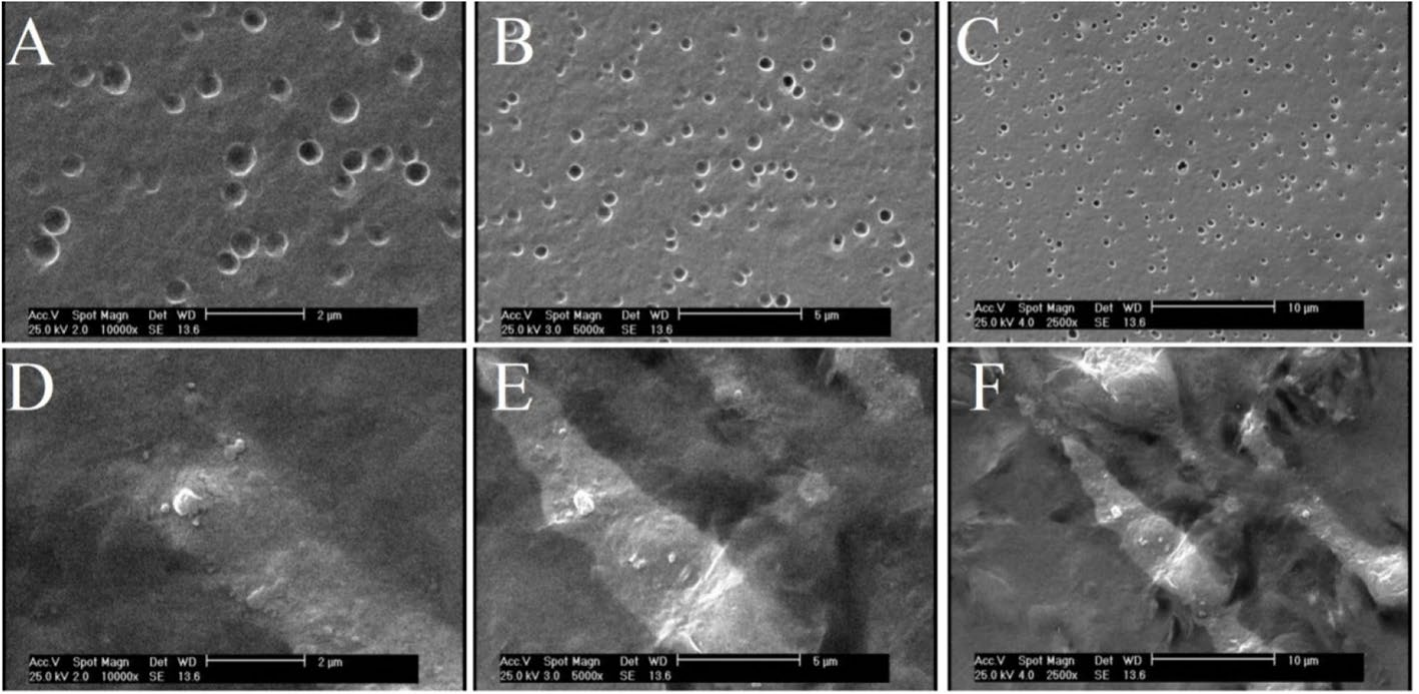
SEM micrographs of PM-CH (**A**, **B**, **C**) and PE-CH (**D**, **E**, **F**). micrographs with a magnification of × 5000 (range 5 μm) and magnification up to × 10,000 (scale 2μm) show submicron particles

Figure 2A-F shows SEM micrographs of PE-CH and PM-CH dried under ambient condition. SEM was used to observe, uniform and homogeneous, the PM. SEM images indicates a spherical morphology of the NPs. The PE-CH and PM-CH NPs size were also evaluated, by DLS indicating, an average size of 302 nm for PM-CH and 378 nm for PE-CH. Our findings demonstrate that PE-CH and PM-CH NPs differ in size and form. This outcome might be the result of variations in the kind and quantity of extract components, such as phenols, oils, waxes, terpenes, and flavonoids. The SEM result shows that, in contrast to PE-CH NPs, which have angular and jagged shapes, PM-CH NPs have a spherical shape

### Encapsulation efficiency (EE%)

The EE% of propolis in chitosan was dependent on the extract's compounds, and the pH of chitosan NPs solutions. Many researches approved that the best EE was achieved by decreasing the pH of the chitosan NPs solution. Total phenol and flavonoid compounds determined by Folin-Ciocalteu assay, aluminum trichloride, and HPLC were used for determination of EE %. Therefore, the EE% were approximately 92.49 and 90.8% for the formulated PM-CH and PE-CH at pH 7, respectively.

### In vitro* release of propolis from the chitosan NPs*

**Fig. 3:**
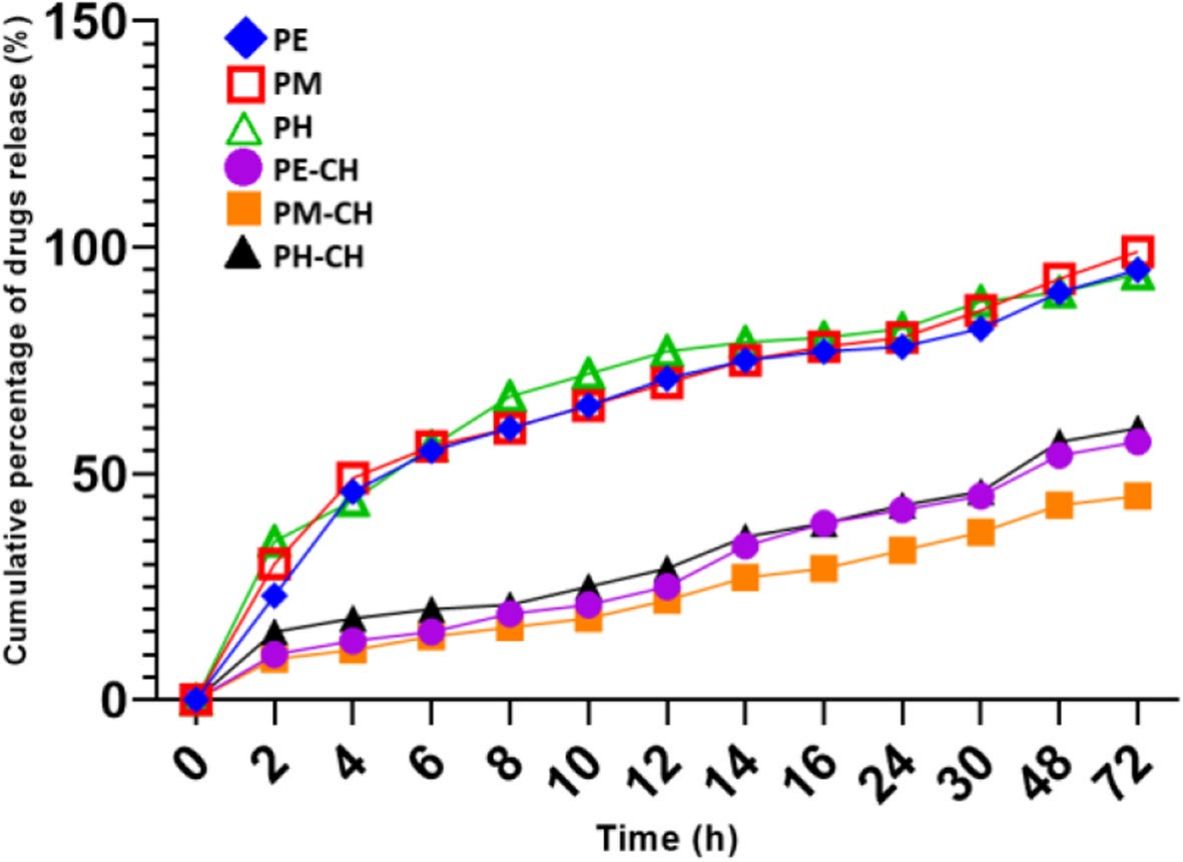
Graph depicting the rate of release of propolis, from the formulation, PE, PM, PE-CH, and PM-CH measured over 72 h

Figure 3 displays the propolis in vitro release profiles from the produced PE-CH, PM-CH, and free PE and PM. PM-CH had a burst release in the initial 2 h, controlled and sustained release over 48 h. After 48 h, the total release was only 43.8%. In contrast, PM exhibited an burst release of 39.2% with in the first 2 h and almost 90% of propolis was released within 48 h. This result shows that PM-CH released propolis in a sustained and controlled manner. The release profile occurs similarly for PE and PE-CH. After 48 h, the total release of PE-CH was only 53.8%. Our result shows that the PE released 37% of the drug for the first 2 h and 90% for 48 h. In general, propolis was released from PE-CH and PM-CH at a slower rate than from PE and PM. According to the first Fick's law was followed by the propolis release from formulations. The release rate of release was closely correlated with the propolis retention in the chitosan NPs that provided sustained release [44].

### Minimum inhibitory concentration (mic) and minimum bactericidal concentration (MBC)

**Fig. 4:**
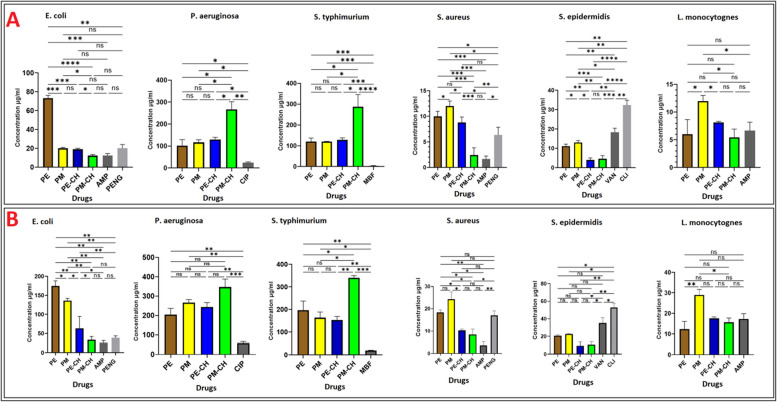
Graph representing MIC(**A**) and MBC(**B**) values for PE, PM, PE-CH, PM-CH against gram-positive and negative bacteria

**Fig. 5:**
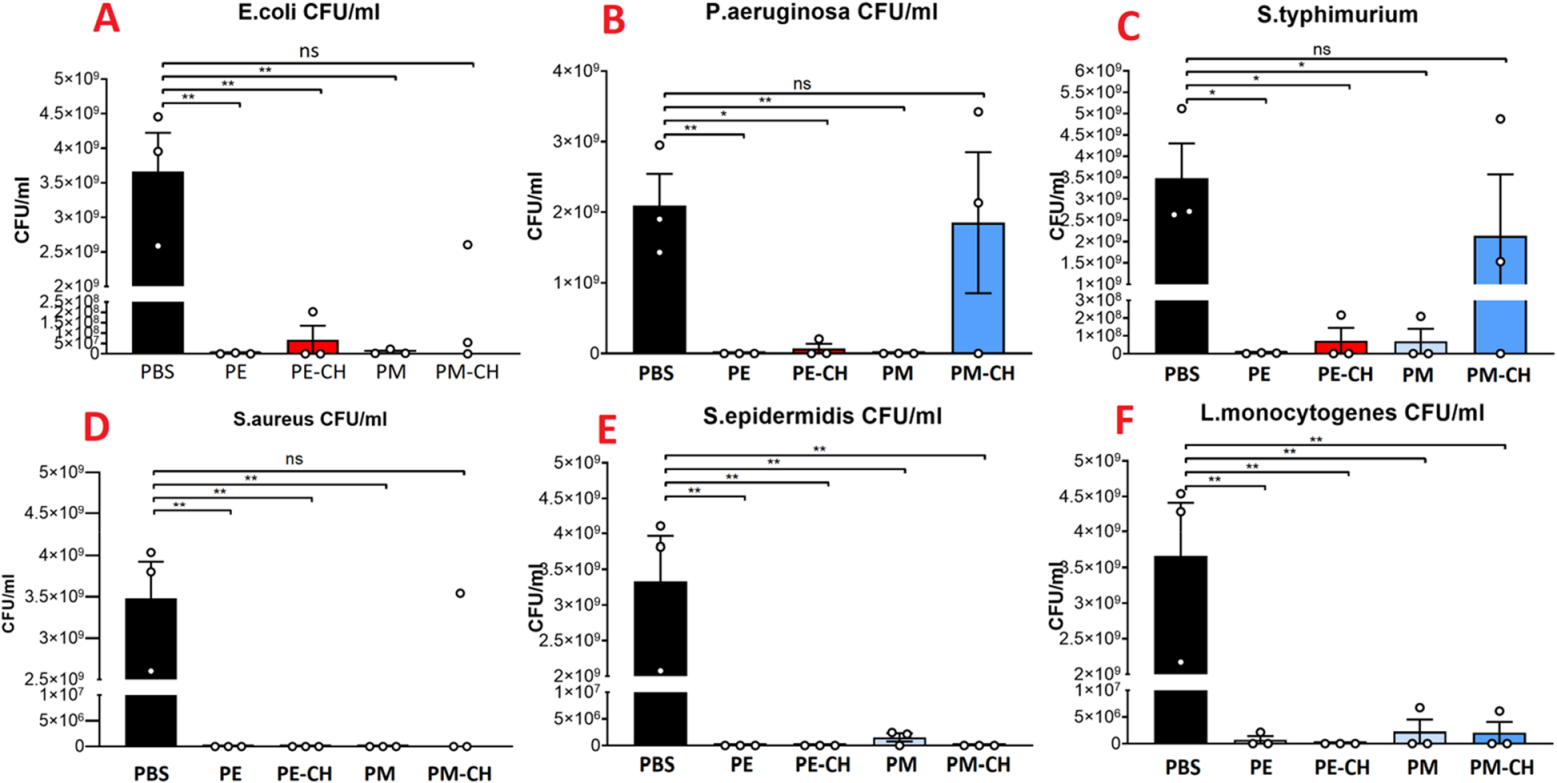
CFU determination of PE, PM, PE-CH, and PM-CH formulation on gram-negative pathogens (**A**: E. coli, **B**: P. aeruginosa **C**: S. typhimurium), and gram-positive bacteria (**D**: S. aureus **E**: S. epidermidis, **F**: L. monocytognes)

In our study, we conducted experiments to test the effectiveness of the PE, PM, PE-CH, and PM-CH against positive and negative bacteria and the result shows a significant reduction in bacterial growth (Table 5, Figs. 4 and 5). MICs values ranged from 2 to 230 µg/ml depending on the microorganisms and drugs. According to our findings, PM-CH was more effective with MIC of 12, 8 µg/ml at reducing E. coli's ability to survive as a negative-gram bacterium. In contrast, the MIC values of PM-CH against gram-positive bacteria, such as *S. aureus*, *S. epidermidis,* and *L. monocytogne,* are 2 and 2.8, and 5, 38 g/ml respective. The outcome demonstrates that PE and PM, in the negative bacteria and positive bacteria, respectively, have a high MIC. Propolis which has been encapsulated with chitosan, such as PM-CH and PE-CH has a low MIC concentration for all tests, indicating that this compound may have a synergistic impact against bacteria. In all assays, PM-CH exhibits a low MIC concentration, except *P. aeruginosa* and *S. typhimurium* with high MIC concentration with better antibacterial properties on the *E. coli, L. monocytognes, S. epidermidis,* and *S. aureus* bacteria*.* In the gram-positive bacteria, *L. monocytognes* with low MIC are sensitive to all propolis compounds. On the other hand, compared to other Gram-positive and Gram-negative bacteria, *P. aeruginosa* exhibits resistance to propolis compounds with high MICs. Additionally, MBC values varied from 3 to 346 μg/ml depending on the microorganisms and propols in various forms. Like the MIC results, the MBC concentration is related to gram-posetive bacteria. The peptidoglycan layer, which covers the outer layer in *E. coli, P. aeruginosa* and *S. typhimurium*, can explains why these organisms are less vulnerable to propolis*. S. aureus* was one of the most vulnerable bacteria overall, and this finding is significant for applaying propolis as a natural antibiotic. According to the HPLC result, Coumaric acid exhibits bacteriostatic activity with a higher concentration in PE and PM extracts, with membrane blabbing similar to the findings of Yoshimasa et al. [45]. Cinnamic acid also has similar antimicrobial activity. Kemperide also has an antimicrobial effect on bacteria that cause skin infections, like S. aureus. As in the previous paper, our findings indicate high concentrations of kaempferide, artepillin-C, drupanin, and p-coumaric acid in PE and PM demonstrated antibacterial and antioxidant activity against *S. aureus, S. saprophyticus, Listeria monocytogenes,* and* E. coli* [15, 46]. PE and PM also contains the flavonoids pinocembrin and apigenin. Research has indicated that isolated pinocembrin possesses antibacterial properties against a variety of bacteria, including *S. mutans, S. aureus, E. faecalis, L. monocytogenes, P. aeruginosa, and K. pneumonia* [47]. *P. aeruginosa, K. pneumoniae, S. enterica, P. mirabilis, and E. aerogenes* are among the Gram-negative bacteria that are inhibited by isolated apigenin [48]. Additionally, there was no bacterial growth seen in the positive control with available antibiotics such as Ampicillin, Penicillin, Ciprofloxacin, Clindamycin, and Vancomycin; however, the chitosan nanoparticles as a negative control without propolis failed to demonstrate activity against the microorganisms examined by the MIC assay.

**Table 5:** MIC and MBC values for PE, PM, PE-CH, PM-CH, and specific antibiotics against Gram-positive and negative bacteria

Bacteria	MIC μg/ml (Drugs)									
	PE	PM	PE-CH	PM-CH	AMP	PENG	CIP	CLI	VAN	MBE
***E.coli***	73	21	19	12.8	10	16	-	-	-	-
***P. aeruginosa***	73	130	140	230	-	-	20	-	-	-
***S. typhimurium***	130	121	129	220	-	-	-	-	-	2
***S. aureus***	11	13	8,37	2	2	5	-	-	-	-
***S. epidermidis***	11.4	13	3	2.8	-	-	-	-	20	35
***L. monocytognes***	5	13	8.37	5.38	-	7	-	-	-	-
	**MBC μg/ml (Drugs)**									
***E. coli***	179	135	47	35	26	36	-	-	-	-
***P. aeruginosa***	218	266	243	346	-	-	58.3	-	-	-
***S. typhimurium***	196.6	163	153	340	19.3	-	-	-	-	-
***S. aureus***	18	24	10.5	8.2	3.5	17	-	-	-	-
***S. epidermidis***	21	22.5	8	11	-	-	-	53	-	-
***L. monocytognes***	15	28	17.7	16	14	-	-	-	-	-

In Fig. 5 (D, E, and F), our result show that the cultures with PE-CH and PM-CH were almost clear of gram-positive bacteria. The CFU determination of these samples showed even better results with complete sterility for almost every of propolis substances. In Fig. 5 (A, B, and C), it is clear that for gram-negative bacteria, all propolis compound substances only worked well for *E. coli*, while *Pseudomonas* showed partial reductions from PM-CH. In addition PM-CH had almost no visible effect on *Salmonella*. Therefore propolis substances worked excellently on three hazardous gram-positive pathogens. As in previous studies, some of them also worked on gram-negative bacteria, but not as promising as on gram-positive pathogens.

These results are also consistent with the results of the prior studies on propolis proving that gram-negative bacteria are more resistant than gram-positive bacteria. This is probably due to fact that gram-negative bacteria create hydrolytic enzymes that disintegrate the propolis' active components [49].

### Biofilm and pre-formed biofilm treatment

**Fig. 6:**
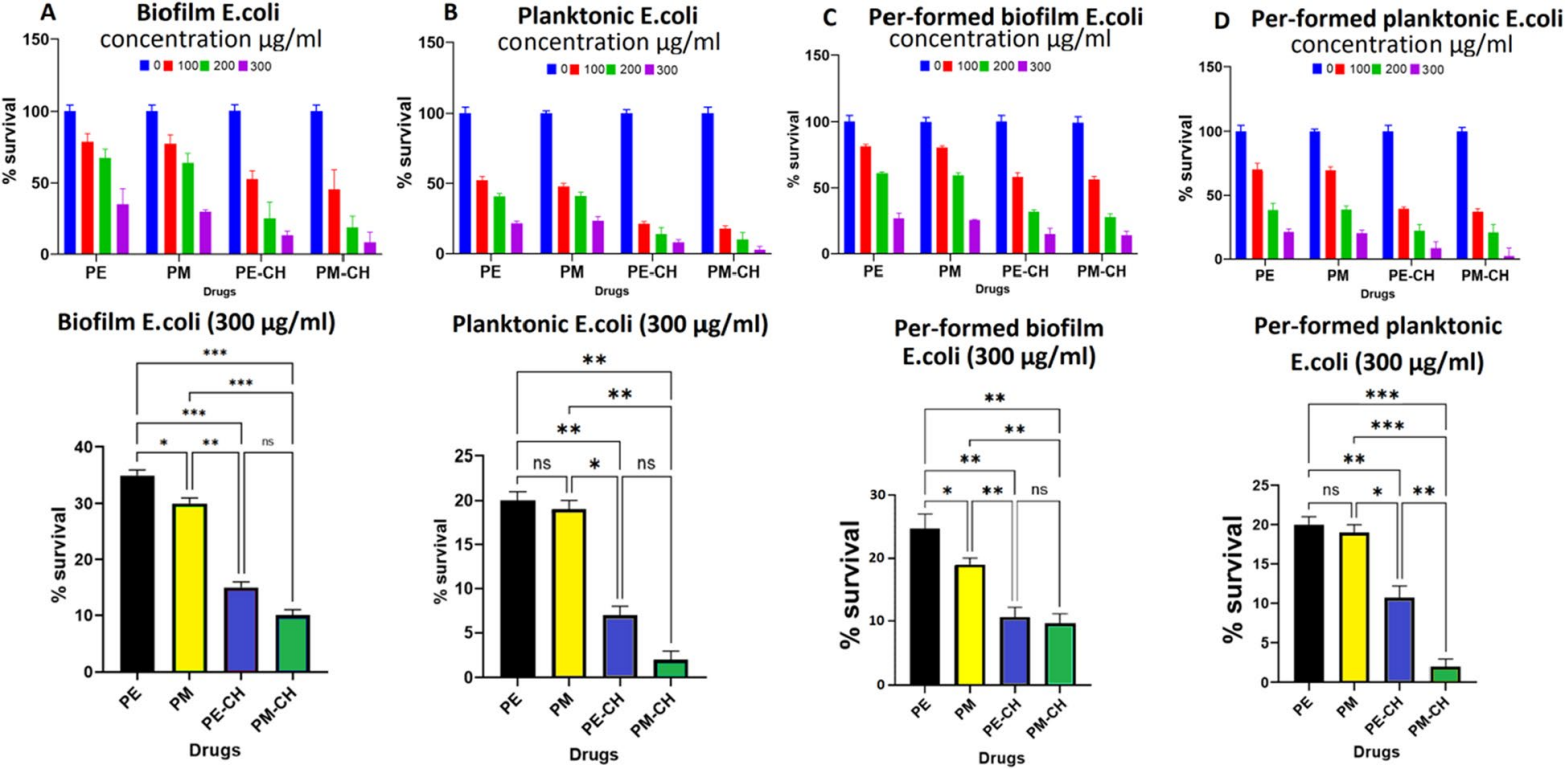
Graphs describing the survival rate of bacteria in biofilm (**A**), planktonic (**B**), pre-formed biofilm (**C**), and pre-formed biofilm planktonic (**D**) from E. coli biofilms treated with PE, PM, PE-CH, and PM-CH. The bottom graph results in 300 μg/mL concentration

**Fig. 7:**
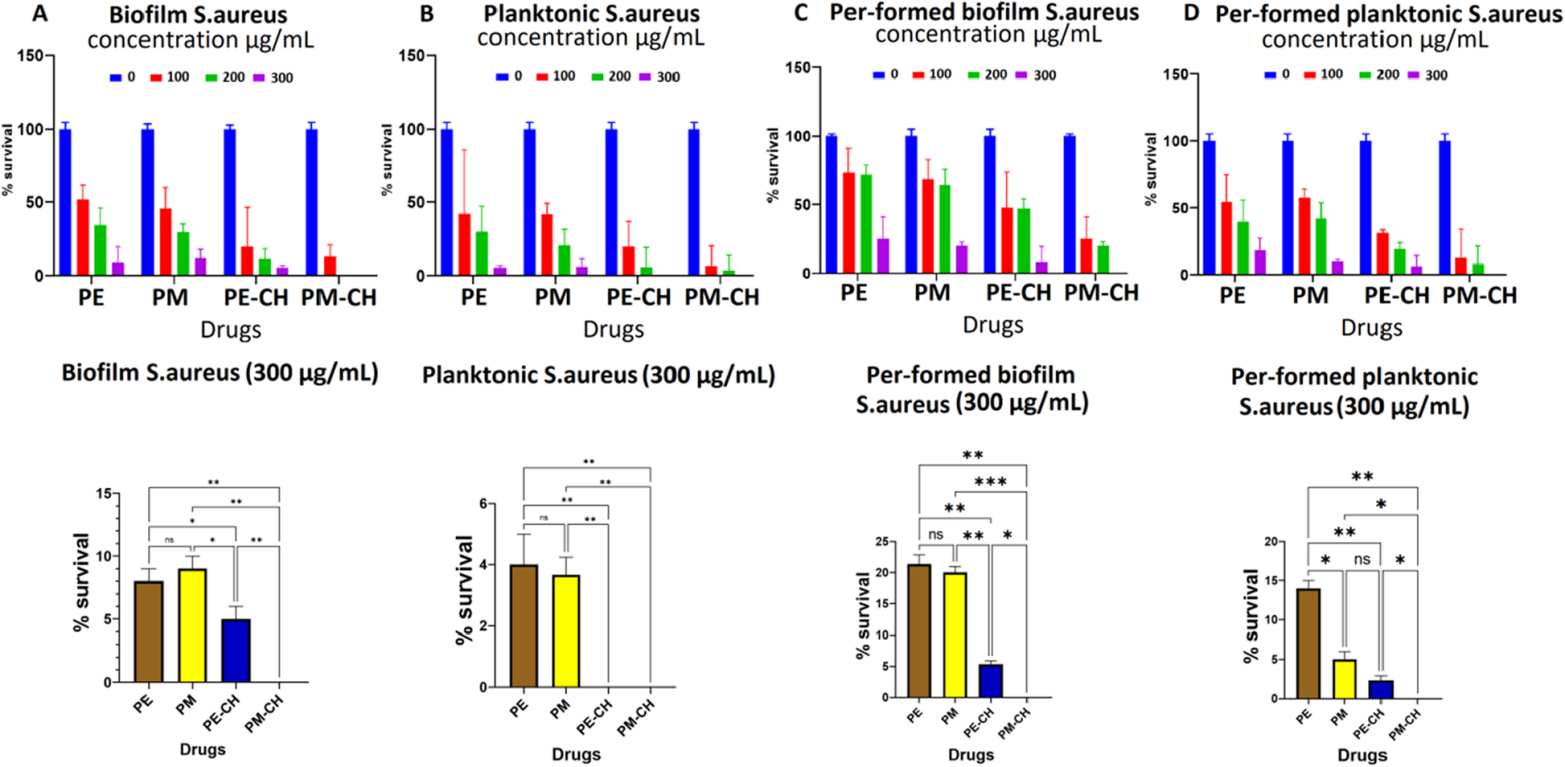
Graphs describing the percentage survival of bacteria in biofilm (**A**), planktonic (**B**), pre-formed biofilm (**C**), and pre-formed biofilm planktonic (**D**) from S. aureus biofilms with PE, PM, PE-CH, and PM-CH. The bottom graph results in 300 μg/mL concentration

**Fig. 8:**
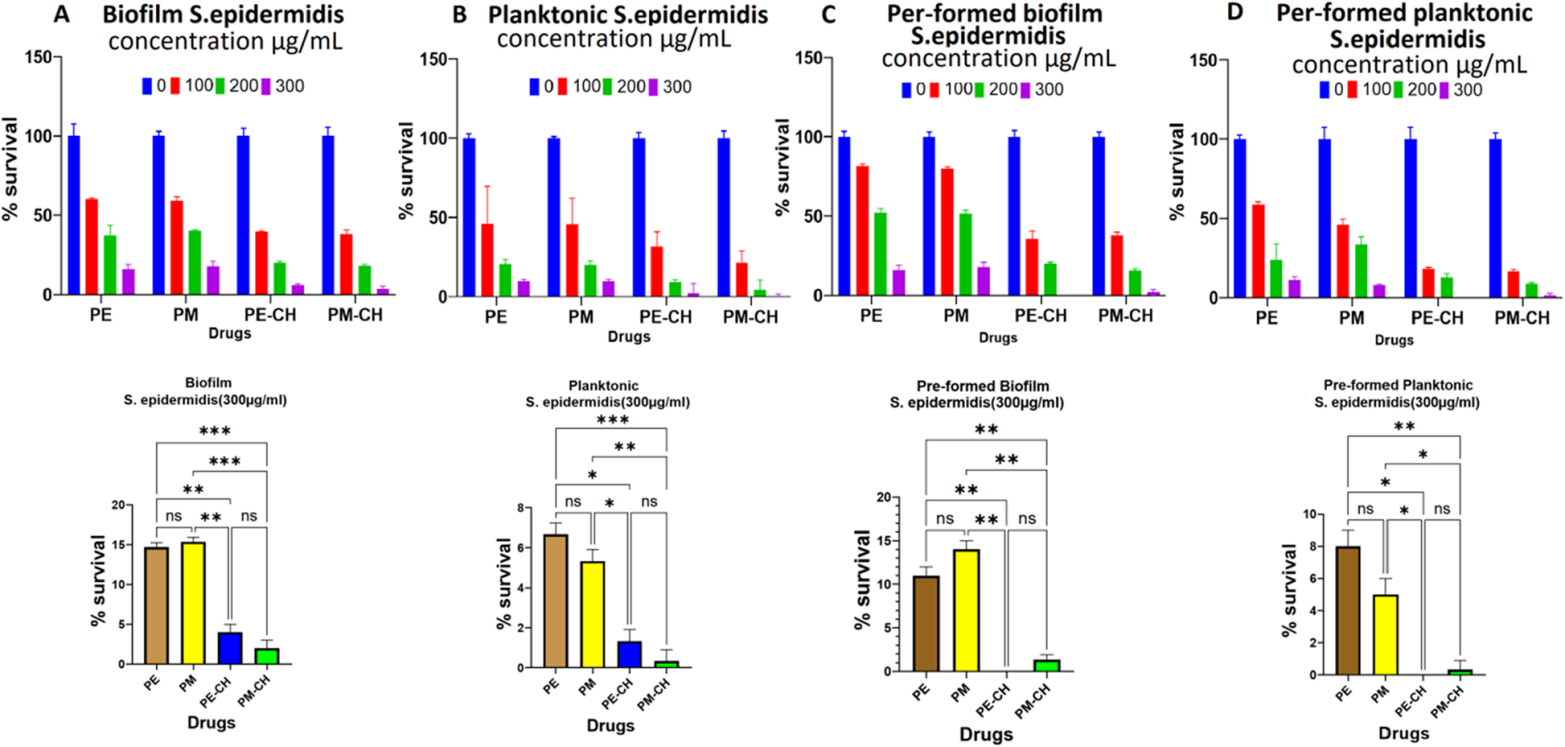
Graphs describing the percentage survival of bacteria in biofilm (**A**), planktonic (**B**), pre-formed biofilm (**C**), and pre-formed biofilm planktonic (**D**) from *S. epidermidis* biofilms treated with PE, PM, PE-CH, and PM-CH. The bottom graph results in 300 μg/mL concentration of PE, PM, PE-CH, and PM-CH

Microorganisms have recently been found to exhibit behaviour that is currently referred to as "group behaviour" or a biofilm perspective. Therefore, to obtain a more thorough understanding of compounds' antimicrobial impacts, it is imperative to investigate their effects on biofilm, pre-biofilm, and planktonic cells. Figures 6, 7 and 8 display the biofilm, planktonic, pre-formed biofilm, and pre-formed biofilm planktonic activity results for each combination. The significant antibacterial properties motivated us to evaluate PE, PM, PE-CH, and PM-CH formulations at different concentrations (100, 200, and 300 μg/ml) again biofilm, planktonic, and pre-formed biofilm bacteria, as well. Although both the PE and PM show a significantly reduced number of *E. coli, S. aureus,* and *S. epidermidis,* our results showed that usage of PE-CH and PM-CH caused a statistically significant greater reduction in the number of *E.coli, S. aureus,* and *S. epidermidis* strains on the biofilm, pre form biofilm and planktonic. Planktonic bacteria displayed greater sensitivity to PE-CH and PM-CH treatment at all concentrations compared to biofilm and pre-formed biofilm assay, in which survival of bacteria was reduced to less than 25–35% with 100 μg/ml, 10–15% with 200 μg/ml, and only 2–5% with 300 μg/ml for *E.coli,* respectively. Our results on *E. coli* bacteria in the biofilm and pre-formed biofilm assay show that PM-CH at 300 μg/ml has a greater effect. Generally, the propolis encapsulation in chitosan has a more substantial impact on Planktonic than on biofilms (Fig. 6). From this, it can be deduced that PE-CH and PM-CH work better at the planktonic, while PM-CH at 300 μg/ml works better against the formation of biofilms.

PE, PM, PE-CH, and PM-CH antibacterial effectiveness against planktonic and biofilm of *S. aureus* bacteria was evaluated. Our result shows the efficacy of PM-CH at 300 μg/ml in which 0% of bacteria survived compared to the PM with only 5–20% of biofilm bacteria survived. Nevertheless, our result shows that when we used PE-CH (300 μg/mL) and pre-formed biofilm treatment, the survival rate decreased to 5%. PM-CH reduced the survival of biofilm, planktonic, pre-formed biofilm, pre-formed biofilm planktonic bacteria by 10%, 5%, 20%, and 5%, respectively, with 100 μg/mL concentration, compared to PE with 50%-70% and PM with 30%-55%. see Fig. 7. This suggests that while PM-CH at 300 μg/ml works better against the formation of biofilms, PE-CH and PM-CH function better at the planktonic stage at the 300 μg/mL.

From the analysis of *S. epidermidis* treatment, biofilm, planktonic, pre-formed biofilm, and pre-formed biofilm planktonic with PE and PM compared to PE-CH and PM-CH were more resistant (Fig. 8). In 300μg/mL concentrations of PE-CH, the result shows that no bacteria survived on pre-formed biofilm, and pre-formed biofilm planktonic bacteria. In 200μg/mL and 300μg/mL of PM-CH, the result shows that nearly 15% and only 3% of biofilm bacteria survived, respectively. In contrast to using the 300μg/mL concentrations of PM propolis extracted, we saw 1–2% of biofilm and pre-formed biofilm bacteria survived respectively. Also, our investigation of the planktonic bacteria from pre formed biofilms displayed high susceptibility to PM-CH (1% survival at 300 μg/mL) compared to PM extracted treatment (4–15% at 300 μg/mL). Also, planktonic bacteria were more sensitive to PE-CH, and PM-CH extracts showed no bacteria survival with 300 μg/mL. At the same concentration, assay with PE and PM resulted in 4% survival of planktonic bacteria. Overall, Bacteria in biofilm and planktonic forms showed more susceptibility to PE-CH and PM-CH compared to PE and PM extracts of propolis. This clearly reveals that NPs are capable of penetrating, due to particle size to pre-formed biofilm bacteria. Also, this may be attributed to the effect of EE% in the case of PM-CH. The similar findings were aslo reported by Ong et al. (2017) who found that propolis extract-loaded chitosan NPs were effective against bacteria in biofilm and planktonic forms [13]. Overall, both biofilms and planktonic bacteria forms show more susceptibility to PE-CH and PM-CH compared to PE or PM.

Every compound and combination exhibited strong inhibitory activity at 24 h, which was the most effective time interval for anti-biofilm activity against bacteria. Three categories can be drawn from the wide range of results for antibacterial drugs: samples with antibacterial and anti-biofilm activities; samples with antibacterial properties but not anti-biofilm activities; and samples with antibacterial properties but not anti-biofilm activities. This procedure implies that substances with anti-biofilm properties but lacking antibacterial properties could function via different channels, like upsetting the structure of the matrix or removing the nutrients required to develop biofilms. An additional hypothesis is that the agents have anti-QS activity, contributing to their ability to disrupt biofilms. According to our findings, there is typically a direct relationship between the capacity to prevent bacterial growth and the inhibition of biofilms. Accordingly, PM-CH exhibits the greatest antibiofilm activity at 24 h, when all combinations and compounds showed bacteriostatic effects of less than 100 μg/ml.

### In vitro* cytotoxicity assay*

**Fig. 9:**
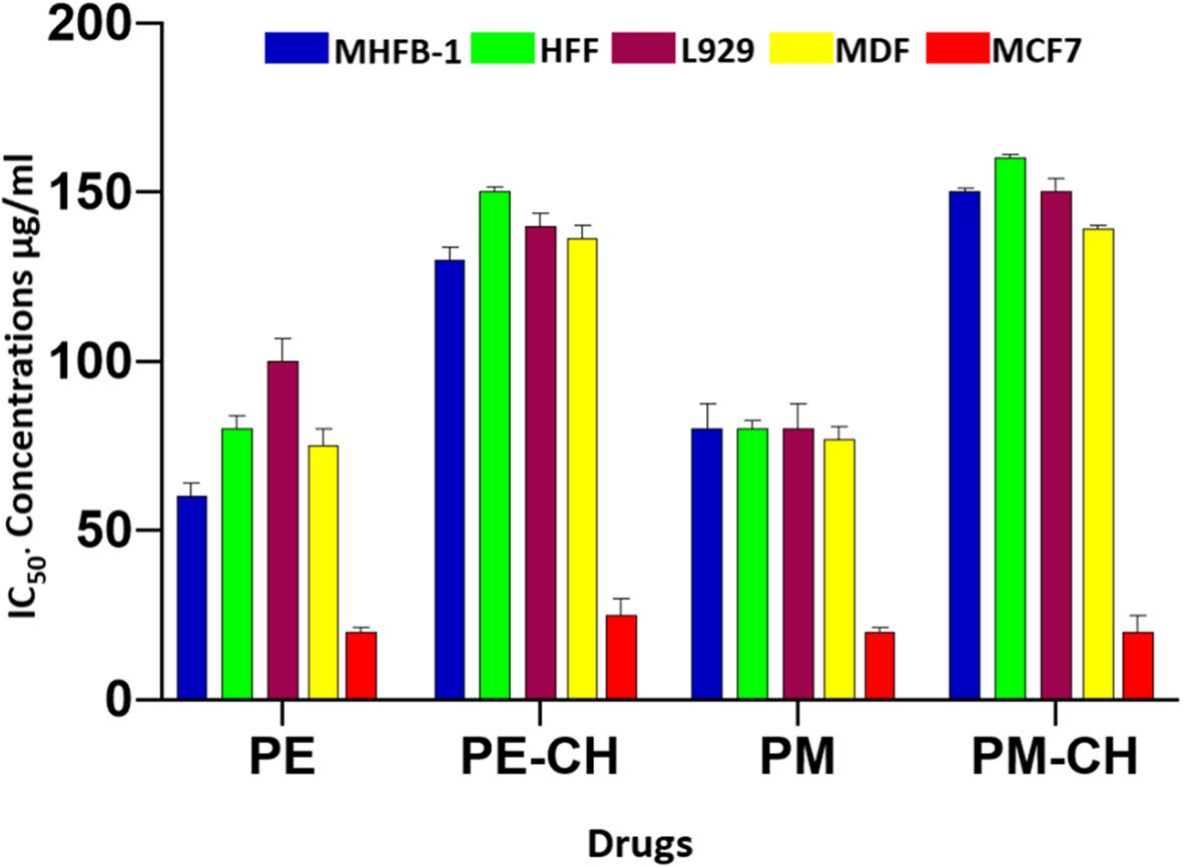
Measurement of cell viability with MTT assay along with Dose–response graph representing the IC50 values (μg/mL) of different PE, PM, PE-CH, and PM-CH formulae

The MTT assay was utilized to assess cytotoxicity because it is a quantitative, accurate, and trustworthy colorimetric approach to establishing cell viability. The cytotoxicity was investigated using MHFB-1, HFF, L929, MDF, and MCF-7 cells which are normal human fibroblasts and cancer cells. Accoding to literature, Propolis has demonstrated a cytotoxic effect on various cancer cell lines [50]. The results of MTT assay are shown in Fig. 9, in which cell viability of experimental groups were compared to the untethered group. The PE, PE-CH, PM, and PM-CH with different concentrations of each sample improved the number of viable cells by determination of IC50 and cell viability. Due to propolis's reliance on different solvents, nanocarriers, and compositions, the reported IC50 values of the compound varied greatly for different cell lines. As shown in Fig. 9, MHFB-1, HFF, L929, MDF cells and MCF-7 cells exhibited statistically significant differences in each other. MCF-7 cells presented lower cell viability. IC50 values of PE, PE-CH, PM, and PM-CH were 60, 130, 75, 150 μg/mL for MHFB-1 cell, 80, 150, 75, 170 μg/mL for HFF cell, 100, 140, 76, 150 μg/mL for L929 cell, 75, 135, 74, 137 μg/mL for MDF, and 20, 23, 20, 21 μg/mL for MCF-7 cell respectively. The cytotoxicity of the cells at the same materials did not significantly differ, according to the results. The cytotoxicity of normal human fibroblasts and cancer cells was found to differ significantly, indicating that propolis and NP are relatively safe. According to the examination of some concentrations, cell viability isn't significantly different for all PE with PM and PE-CH with PM-CH groups. These results show that propolis at a concentration of up to 150 μg/mL could promote the proliferation of normal cells. The difference was observed between IC50 values of PE, this confirmed the relative of propolis concentration dependent. In addition, propolis encapsulation into chitosan NPs resulted in a high IC50 for normal cells. All PE, PM, PE-CH, and PM-CH substances are highly cytotoxic to MCF-7 cells at 20 μg/mL concentrations. This result is in accordance with previous studies, which indicated that some antioxidant compounds of propolis cause increased cell proliferation. The findings, which are shown in Fig. 9, demonstrate that cell viability fell in a dose-dependent manner when exposed to rhe teasted chemical and that it was marginally reduced at higher doses. Also, the results showed no discernible difference in the cytotoxicity of the tested compounds.

Similar to the obtained, previous reports, IC50 for different normal and cancer cells substantiated the variety in the observed cell viability. Frozza et al., reported hydroalcoholic extracts of propolis with IC50 values between 14.4 to 150 μg/mL for normal and human laryngeal epidermoid carcinoma cell lines [51, 52]. Cheah et al. evaluated the cytotoxic dose for tumor and normal cells 30 and 50 g/ml for Hct15 cell [53]. Iranian propolis was tested for their impact on the vitality of L929 from 25 to 800 g/ml, and they did not exhibit any cytotoxicity at concentrations below 200 g/ml; however, at 400–800 g/mL, the viability was compromised to 50% [54].

## Discussion

Propolis has different antimicrobial mechanisms, including inhibition of cell division, disruption of cell wall integrity, inhibition of bacterial motility, surface-exposed adhesions and polypeptides and cell membrane enzymes, bacteriolysis, blocking of ion channels, Inhibition of electron by capturing electrons and protein synthesis inhibition [55] change surface protein three dimensional (3D) structure [56]. With the latter mechanism, the microbial proteins and propolis polyphenols create ionic and hydrogen interactions change the proteins' 3D structure and functions [57]. In multiple studies it was found that Gram-positive bacteria were more susceptible than Gram-negative bacteria [58] which was attributed to specific structure of the outer membrane such as a multilayered membrane in Gram-negative than a single layered membrane in the Gram-positive bacteria [59]. Also, Gram-negative bacteria create hydrolytic enzymes that disintegrate the propolis' active components [49, 60]. There are many different reports on the characterization of propolis and evaluation of its anti-bacterial effect against many bacteria such as *S. mutans* [61], *S. epidermidis* [56], *Enterococcus faecalis* [13] *P. aeruginosa, S. typhi, E.coli, S. aureus, B. subtilis, Enterococcus sp, and Candida spp* [62]. Additionally, the antibacterial efficacy of propolis from various parts of the world was comapred. These reports mentioned that the mechanism of propolis activity depends on the synergy between compounds in propolis, the effect of extracting methods and their interference with microbes. The different ranges of MIC and MBC for various solvents, including alcohol, water, DMSO, DCM, Hexane, and supercritical fluid have been recorded [5]. We conducted a comparison between the composition and biological activities of propolis extracts and a nonalcoholic a substitute solvent mixture, such as Metylal, which we used as a green solvent. In the literature, there are references to other nano formulation ns that contain propolis extracts, but they have distinct formulations and uses [63–65]. The size, shape, and surface charge of nanoparticles (NPs) as well as how they interact with biological systems can influence NP behavior and ultimately determine their toxicity. Also, encapsulation inside chitosan NPs offered a better dispersability, and prolonged release time of propolis. Drugs encapsulated in nano particles carriers with surface charges are resistant to enzymatic degradation [66]. We hypothesize that extracting propolis with Methylal and formulating it into nano-sized chitosan will improve its antibiotics effects and thus it’s cytotoxicity. We found that Metylal is a excellent solvent for the most active ingredients of propolis, including hydrophilic and lipophilic compounds. The PM-CH formolations revealed that a chitosan polymeric system does not have a negative impact on the stability, potency, or biological activity of flavonoids and phenols in propolis extract. Therefore, in this study, we have chosen 0.2% w/v as an ideal concentration of chitosan. Only physical interactions between the propolis extract, and the chitosan were observed; no significant chemical interactions were evident. Due to positive zeta potential of some molecules like chitosan, enhances drug delivery by facilitating adherence to the negatively charged cell membrane. The kinetics of drug release from NPs should therefore be a key component of their design and a trait that is checked for quality [67]. Chitosan with a positive surface charge, has interacted with negatively charged bacteria surface, thereby resulting in the cell surface permeability and then inhibiting of bacterial growth with bind to the anionic bacteria and increase their zeta potential [20]. The results of this investigation agree with that reported by Teik and co-worker which indicated that decreasing and increasing the chitosan concentration below 0.2% w/v resulted in clumping. These authors also confirmed that chitosan solution above 0.5% w/v lead to the formation of large, aggregated nanoparticles with aparticle size above 400 nm [13]. In general, propolis was released from PE-CH and PM-CH at a slower rate than from PE and PM. Consequently, chitosan NPs released the propolis continuously and under control, preventing changes in the release rate. As a consequence, both Gram-positive and Gram-negative strains showed significant antibacterial activity for Middle Eastern propolis [58]. In the present study, PE, PM, PE-CH and PM-CH inhibited the growth of Gram-negative bacteria such as *E. coli, P. aeruginosa*, and *S. typhimurium* with MIC values ranging from 12.8 to 230 μg/ml. Also, PE, PM, PE-CH and PM-CH inhibited the growth of gram-positive bacteria such as *S. aureus, S. epidermidis,* and *L. monocytognes* with MIC values ranging from 2 to 13 μg/ml. The outcomes were consistent with another study on Gram-positive bacteria, which had MIC values ranging from 3 to 100 g/ml [68]. The majority of Gram-positive bacteria create extracellular thin coatings of glucans, which, when present with sucrose, cause adhesion and biofilm development. The succeeding bacterial population produces biofilms after the initial bacterial adhesion to a particular surface [69]. Both pre-formed biofilm and biofilm assay are important methods for evaluating the effectiveness of antimicrobial agents against biofilms, which are known to be more difficult to eradicate than planktonic bacteria. Since propolis extracted with Methylal has a high antioxidant activity and the potential to suppress bacterial growth, it was used to create the nano emulsion. The antioxidant activity may be caused by phenolic substances [70]. Chemically, phenols are composed of an aromatic ring that is linked to one or more hydrogenated substituents, along with their derivatives. While all propolis extract methods lower the membrane potential of bacteria, our result shows that chitosan-propolis Methylal extracted NPs (PE-CH and PM-CH) have a great effect against suspension planktonic as well as biofilm of *E. coli, S. aureus*, *S. epidermidis* bacteria. The results of MIC and MBC for PE and PM activity demonstrate that compounds like phenolic acids, esters, flavonoids, and terpenes are likely to react with light, air, water, and other extrinsic elements, promoting rapid degradation or inactivation of biological activity. However encapsulation of propolis inside in chitosan NPs could prevent degradation and deactivation of the propolis extract. Our data shows that lower PE-CH and PM-CH concentrations are sufficient to kill planktonic bacteria compared to biofilm and pre-formed biofilm established by *S. aureus* and *S. epidermidis* due to small particle size and the positive zeta potential, has better antibacterial efficacy as compared to PM and PE extracts which enables them to penetrate in to the biofilm. Contrary to our results obtained with nanoparticles of propolis, although PM-CH could reduce *E. coli* in biofilms by 98–100% (at concentration of 300μg/ml), at the same concentration, only 65–80% of bacteria in pre-formed were eradicated. Similar results were obtained for the other positive bacteria. The DPPH assay displayed good antioxidant activity, with inhibition values of 43% for PM-CH and only 36% for the PE-CH sample. Our findings indicate that propolis's high potential for antioxidants in nature makes it a potential functional drug and food. There is a strong correlation between propolis's antioxidant capacity and its phenolic compounds, minor components, amino acids, flavonoids, terpenes, steroids, aldehydes, and ketones, which are the main sources of propolis's antioxidant capacity (Tables 2 and 3). Propolis's antioxidant properties are thought to be mediated by a variety of mechanisms, including metallic ion chelation, hydrogen donation, free radical sequestration, and serving as a substrate for radicals like hydroxyl and superoxide. Additionally, it has been proposed that the organic acids (Tables 2 and 3) in propolis enhance the effects of flavonoids and, via metal chelation, contribute to antioxidant activity. It should be noted that flavonoids are more easily oxidized the more hydroxyl groups they contain. For this reason, maybe nanoencapsulation of propolis can reduce the oxidation of flavonoids to have a lasting and more effect [40]. Natural-derived propolis has cytotoxic effects on several cell lines. For various cell lines, it was shown that the reported IC50 values of propolis varied greatly, which might be explained by the fact that the ingredients of propolis depend on its geographic origin, bee type, plant pollen, etc. Due to propolis's reliance on different solvents, nanocarriers, and compositions, the reported IC50 values of the compound varied greatly for different cell lines. The cytotoxice results showd a discernible difference between IC50 values of PE and PM with PE-CH and PM-CH, confirming the relative safety of pure propolis and encapsulation form. Similarly, great doses of PE-CH and PM-CH showed comparable cellular viability of healthy cells but compromised the cancer cells. Overall, the choice of solvent, extraction method, and choice of suitable nano carrier should be carefully considered based on the targeted properties of the propolis material and the biological effects of the extract.

## Conclusions

In this study, we have substantiated that the formulation based on chitosan NPs encapsulated propolis, and the extract of propolis with Methylal have great therapeutic effecieces toward combating bacterial biofilms. Our MIC, MBC, and biofilm assay results like previous studies showed that Gram-negative bacteria are more resistant than Gram-positive bacteria. The synergistic effect of chitosan NPs and propolis has amplified the the antimicrobial effeciencies. Our MIC, MBC, and all biofilm assay results are in agreement to those from propolis-producing Middle Eastern nations like Turkey and Oman which indicates the same plants were the food source of bees use to make propolis. Our research has shown that the Methylal extract of the propolis has a high total flavonoid and phenolic contents. In comparison to free propolis, the release profiles of PE-CH and PM-CH demonstrated that propolis compound release from NPs is slower, which sustained the release of phenols and flavonoids against destruction. Considering these factors, MIC, MBC, and all biofilm assays reports reports on the successful extraction of propolis with metylal and successful encapsulation of propolis in chitosan, can be possible great substitutes for industrial antibiotics. Early indications that both PE-CH and PM-CH compounds have great effect against some bacteria that have resistance to the routine antibiotics. Both PE-CH and PM-CH had a favourable effect on the cell culture and increased the quantity of viable cells in terms of cell viability. Propolis and NP were found to be relatively safe, as evidenced by the significant difference in cytotoxicity between cancer cells and normal human fibroblasts. A final general protocol's development may be challenged by the fact that various experiments produce different results from each other. Furthermore, at higher concentrations than indicated in the manuscript, methylal can be dangerous for the researcher if safety precautions are not taken. This suggests that the extract may have potential antioxidant properties, which could be beneficial for various healthcare applications, including wound healing, natural antibiotics, cosmetic uses, and cancer therapy.

### Supplementary Information

**Supplementary Material 1**.

## Data Availability

All data generated or analyzed during this study are included in this published article [and its supplementary information files].

## Abbreviations

PE: Propolis extract with Ethanol
PM: Propolis extract with Methylal
PE-CH: PE encapsulation with chitosan nanoparticles
PM-CH: PE encapsulation with chitosan nanoparticles
HPLC: High-performance liquid chromatography
GC-MASS: Gas chromatography–mass spectrometry
PDI: Poly dispersity index
DLS: Dynamic Light Scattering
SEM: Scanning electron microscopy
MTT: Tetrazolium-based assay
DPPH: 2,2-Diphenyl-1-picrylhydrazyl
FTIR: Fourier-transform infrared spectroscopy
MIC: Minimum Inhibitory Concentration
MBC: Minimum Bactericidal Concentration
