# Supplementary material for: Encapsulation of propolis extracted with methylal in the chitosan nanoparticles and its antibacterial and cell cytotoxicity studies
Source: BMC Complement Med Ther. 2024 Apr 19;24:165. doi: 10.1186/s12906-024-04472-8 (PMC11027551; doi:10.1186/s12906-024-04472-8)
Supplement: Supplementary file 1 — Supplementary Material 1. [file 12906_2024_4472_MOESM1_ESM.docx]

**Supporting Information**

**Encapsulation of Propolis Extracted with Methylal in the Chitosan Nanoparticles and its Antibacterial** **and Cell cytotoxicity studies**

**Akbar Vaseghi^a^, Reza Ashrafi Parchin^b^, Kosar Rezaee Chamanie^c^, Marc Herb^d^, Hajar Maleki^e,f^, Majid Sadeghizadeh^g^***

1. *Department of Nanobiotechnology, Faculty of Biological Sciences, Tarbiat Modares University, Tehran, Iran*
2. *Excir Faravaran Sabalan Company, Ardabil Science and Technology Park, Ardabil, Iran*
3. *Department of Biology, Faculty of Science, University of Guilan, Rasht, Iran*
4. *Institute for Medical Microbiology, Immunology and Hygiene, Faculty of Medicine and University Hospital Cologne, University of Cologne, 50935 Cologne, Germany*
5. *Department of Chemistry, Institute of Inorganic Chemistry, University of Cologne, Cologne 50939, Germany*
6. *Center for Molecular Medicine Cologne, CMMC Research Center, Cologne 50931, Germany*
7. *Department of Molecular Genetics, Faculty of Biological Sciences, Tarbiat Modares University, Tehran, Iran*

Corresponding author contact: Majid Sadeghizadeh, Prof.

Department of Genetics,Faculty of Biological Sciences,Tarbiat Modares University, Jalal AleAhmad st, Tehran, Iran.

P.O.Box: 14115-154

Tel: +98 21 82884409

Fax: +98 21 82884484

E-mail: sadeghma@modares.ac.ir

Current address: Department of Molecular Genetics, Faculty of Biological Sciences, Tarbiat Modares University, Tehran, Iran

**
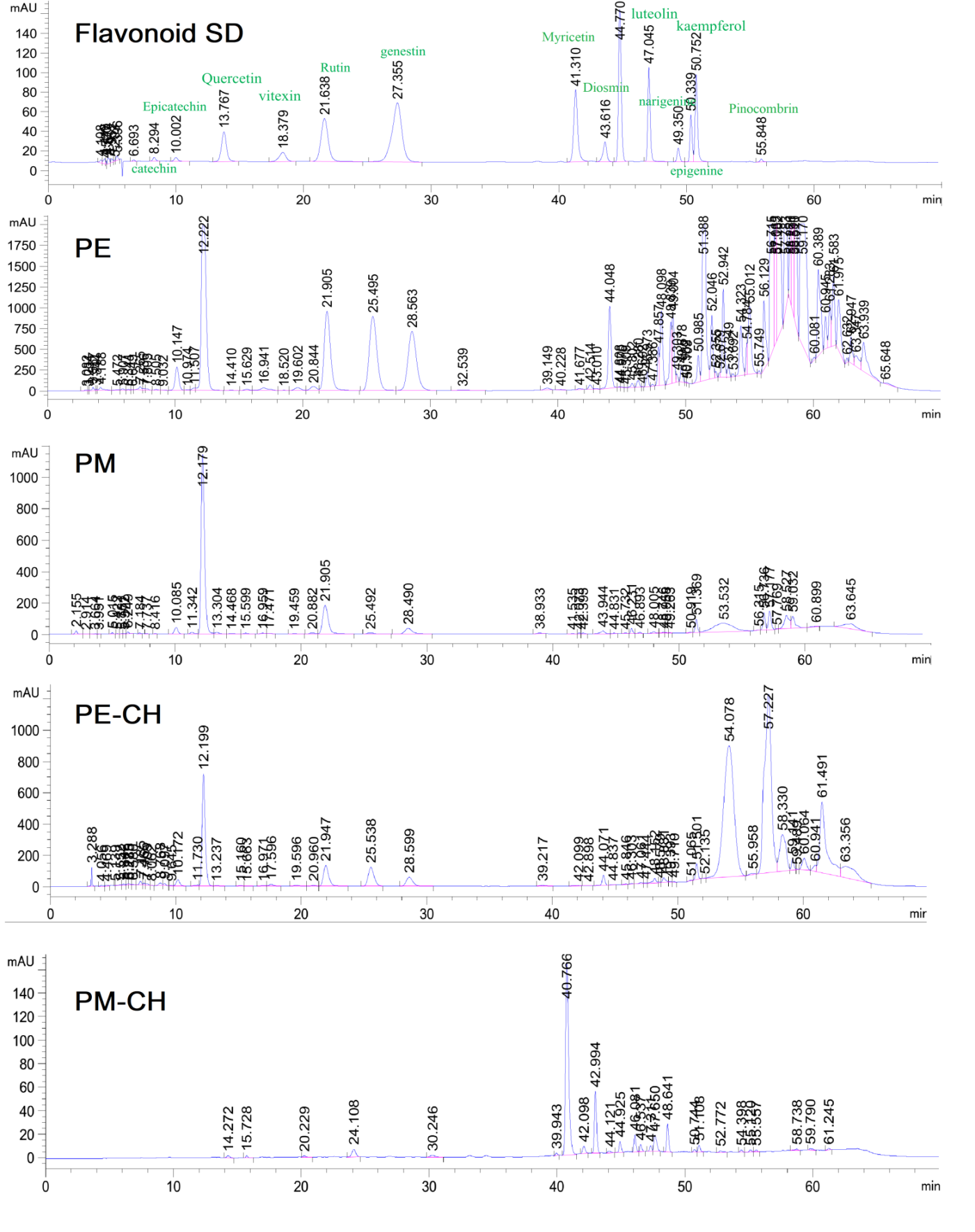
Fig. S1** HPLC chromatograms depicting the elution profile of standard flavonoid compounds and propolis extracted. Chromatographic profile of the flavonoid Standard, Ethanol (PE), Methylal (PM), PE-CH and PM-CH depicting the presence of the flavonoids represented by the peaks marked with arrows.


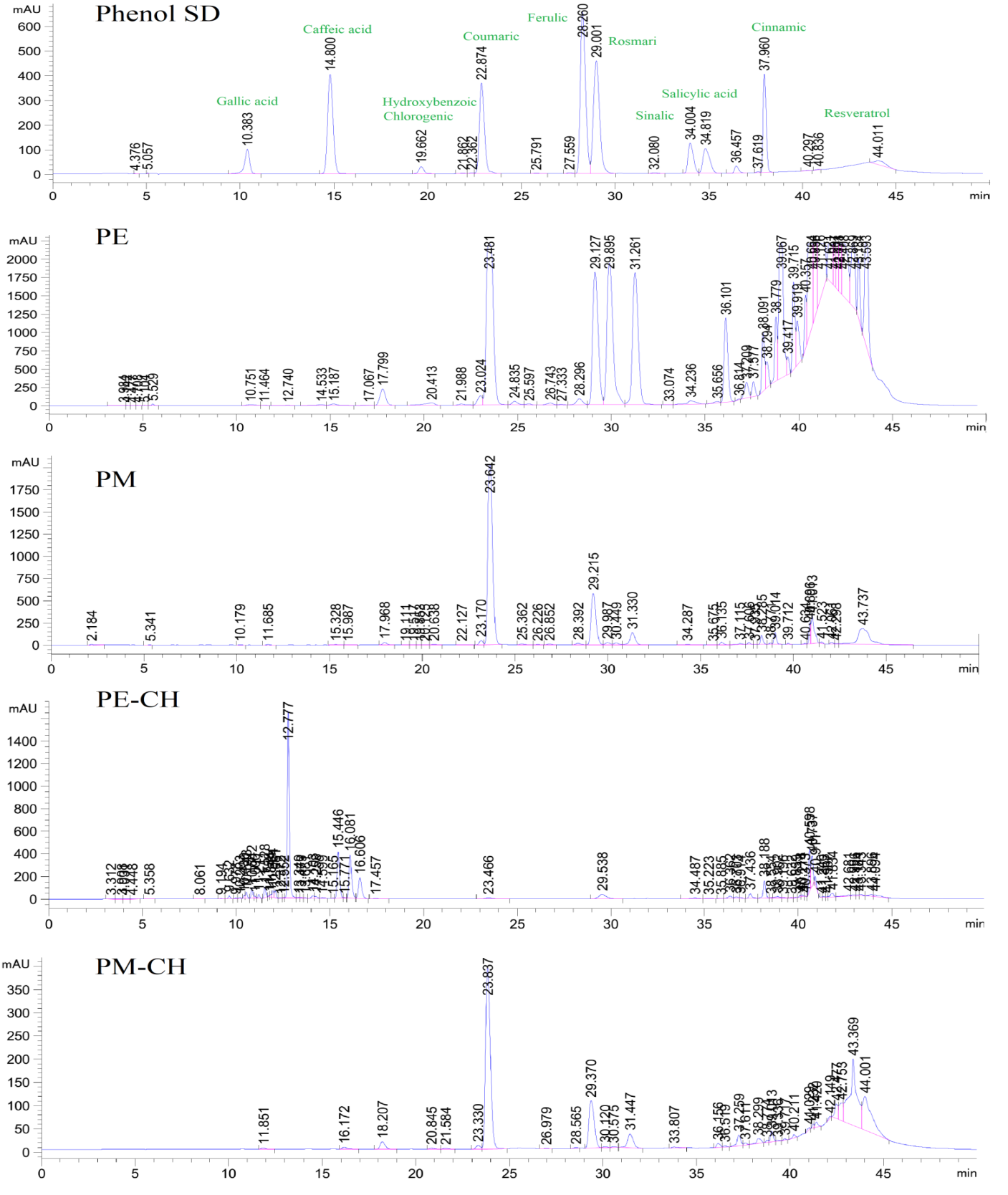


**Fig. S2** HPLC chromatograms depicting the elution profile of standard phenols compounds and propolis extracted. Chromatographic profile of the phenol Standard, Ethanol (PE), Methylal (PM), PE-CH and PM-CH depicting the presence of the phenol represented by the peaks marked with arrows.


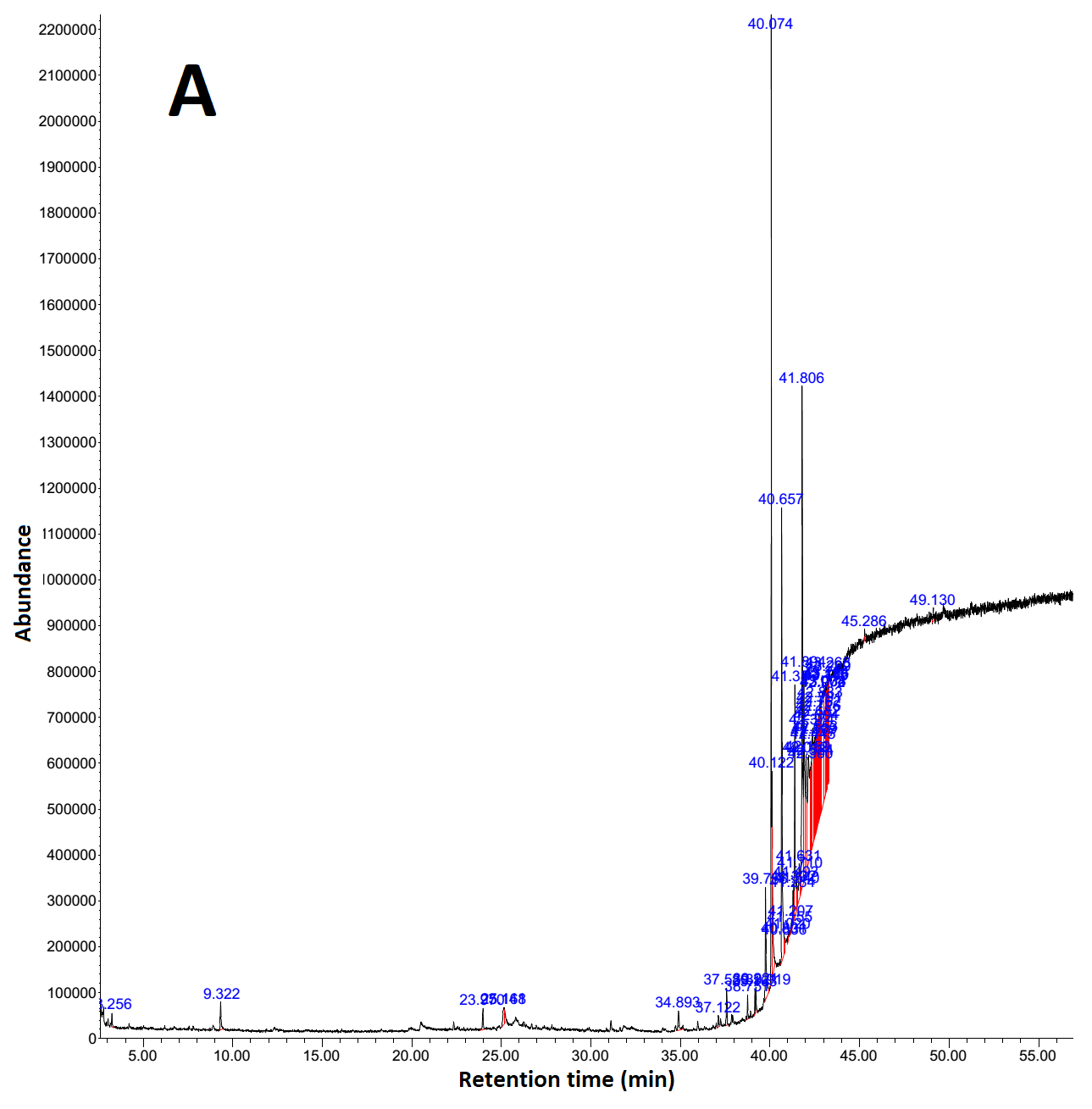


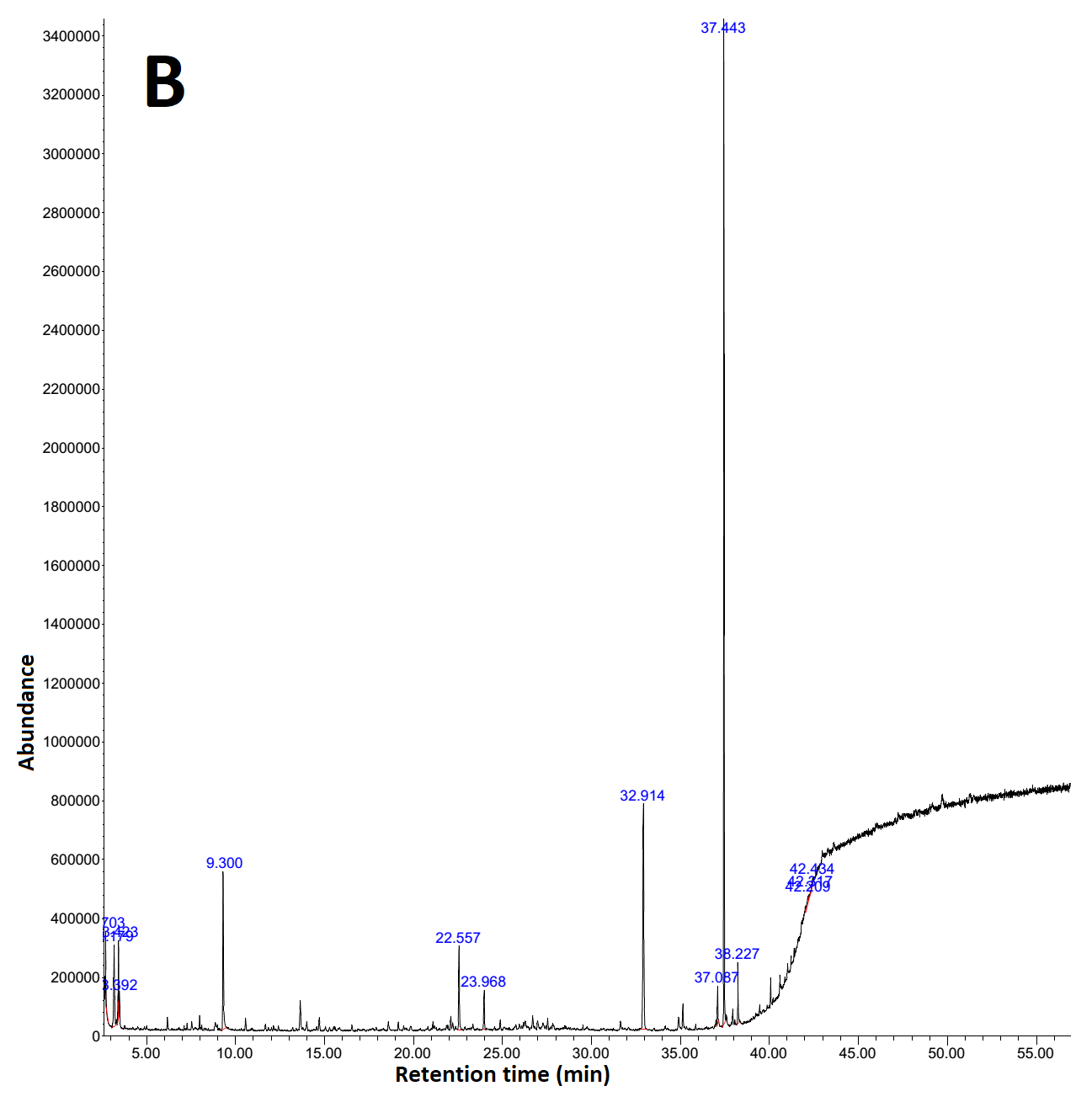


**Fig. S3** The chromatogram of the PE (A) and PM (B) according to GC–MS analysis.

**
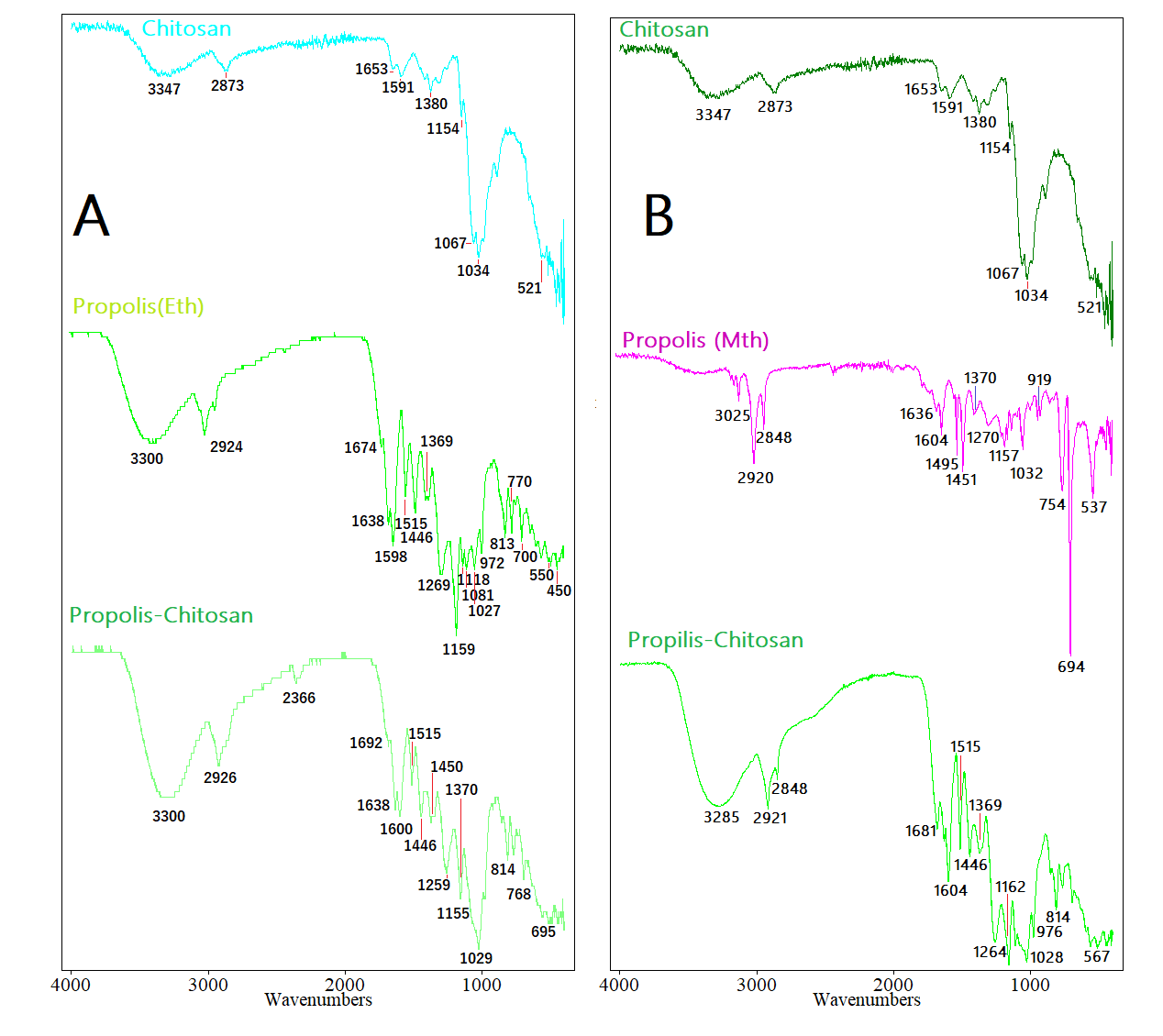
**

**Fig. S4** FTIR spectra of chitosan, PE-CH, and PM-CH nanoparticles loaded with PE, PM in different compositions. Comparative FTIR spectra of chitosan and Ethanol extract of propolis (A) and Metylal extract of propolis (B).

**
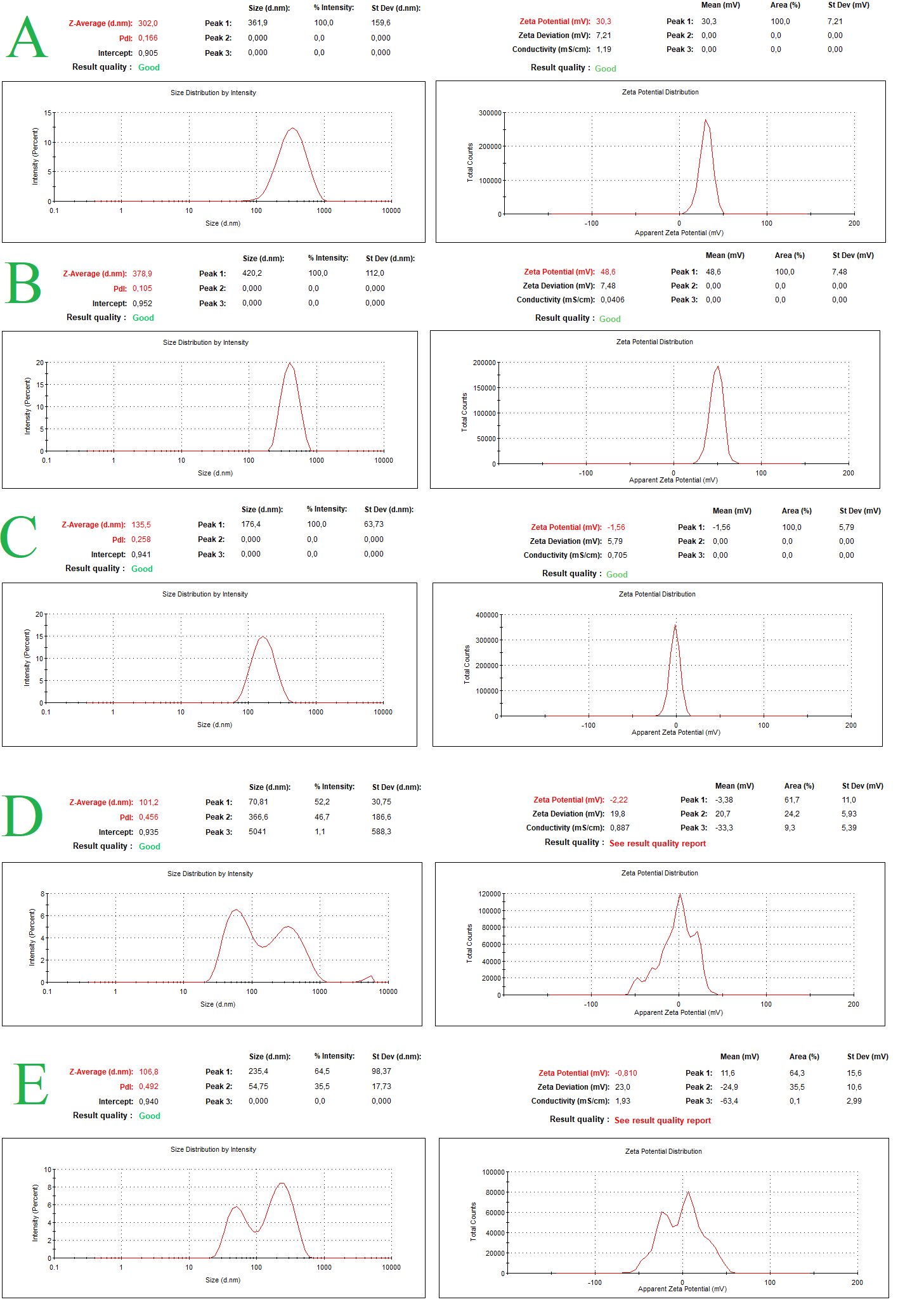
**

**Fig. S5.** The DLS results indicating nanoparticles size, zeta potential, and polydispersity index (PDI) of PM-CH (A) and PE-CH(B)
